# Supplementary material for: A universal glycoenzyme biosynthesis pipeline that enables efficient cell-free remodeling of glycans
Source: Nat Commun. 2022 Oct 24;13:6325. doi: 10.1038/s41467-022-34029-7 (PMC9592599; doi:10.1038/s41467-022-34029-7)
Supplement: Supplementary file 1 — Supplementary Information [file 41467_2022_34029_MOESM1_ESM.pdf]

Supplementary Information

A universal glycoenzyme biosynthesis pipeline that enables efficient cell-free remodeling of glycans

Thapakorn Jaroentomeechai[1], Yong Hyun Kwon[1], Yiwen Liu[1], Olivia Young[1], Ruchika Bhawal[2], Joshua D. Wilson[3], Mingji Li[1], Digantkumar G. Chapla[4], Kelley W. Moremen[4], Michael C. Jewett[5], Dario Mizrachi[6], and Matthew P. DeLisa[1,2]*

[1]Robert F. Smith School of Chemical and Biomolecular Engineering, Cornell University, 120 Olin Hall, Ithaca, NY 14853, USA

[2]Cornell Institute of Biotechnology, Cornell University, Ithaca, NY 14853, USA

[3]Glycobia, Inc., 33 Thornwood Drive, Suite 104, Ithaca, NY 14850, USA

[4]Complex Carbohydrate Research Center, University of Georgia, Athens, GA 30602, USA

[5]Department of Chemical and Biological Engineering, Northwestern University, 2145 Sheridan Rd Technological Institute E136, Evanston, IL 60208-3120, USA

[6]Department of Physiology & Developmental Biology, Brigham Young University, Provo, UT 84602, USA

*Address correspondence to: Matthew P. DeLisa, Robert Frederick Smith School of Chemical and Biomolecular Engineering, Cornell University, Ithaca, NY 14853. Tel: 607-254-8560; Email: md255@cornell.edu

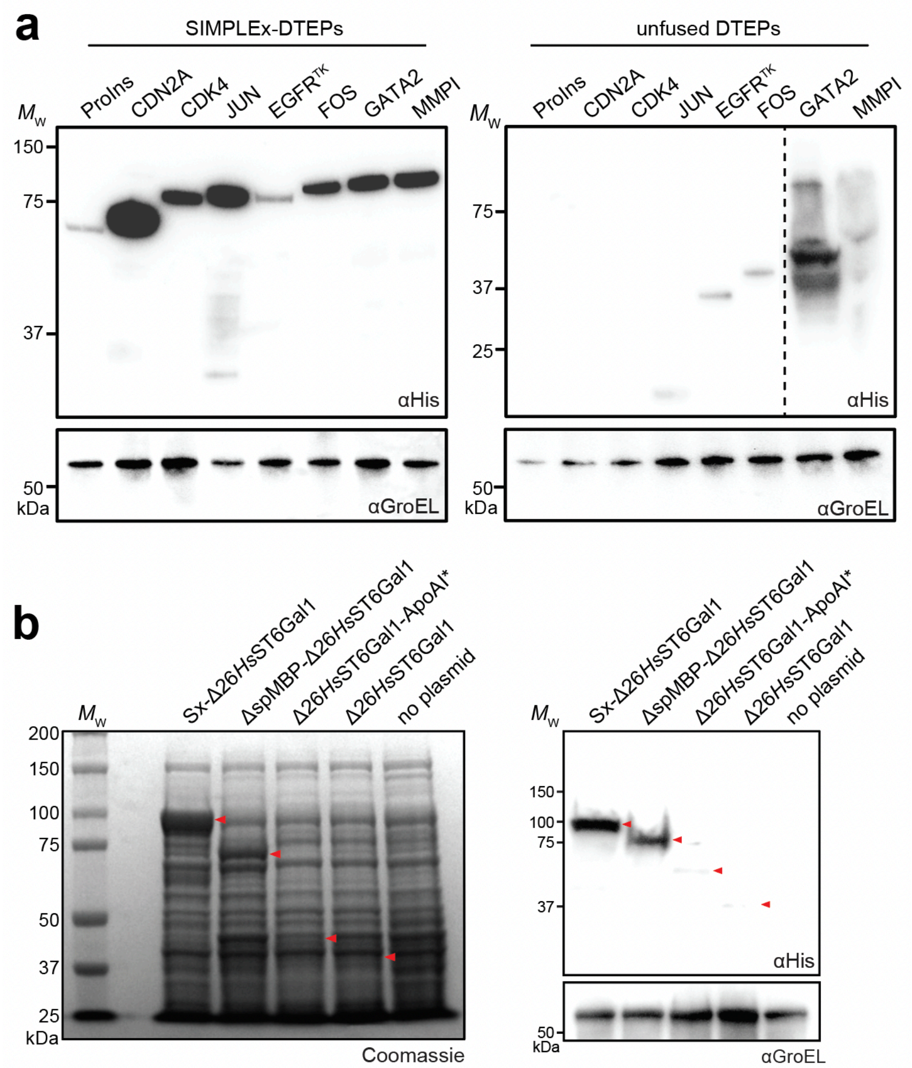

Supplementary Figure 1. SIMPLEx architecture promotes soluble expression of difficult-to-express proteins (DTEPs). (a) Immunoblot analysis of the soluble fraction prepared from *E. coli* cells expressing human-derived DTEPs as SIMPLEx fusions (left panel) or as unfused proteins (right panel). *E. coli* BL21(DE3) was used to express human transcription factors (GATA2, JUN, and FOS), human cyclin-dependent kinase 4 (CDK4), and human cyclin-dependent kinase inhibitor 2A (CDKN2A), while SHuffle T7 Express *lysY* strain was used to express human epidermal growth factor receptor tyrosine kinase domain ($EGFR^{TK}$), human matrix metallopeptidase 1 (MMP1), and human proinsulin (ProIns). Human gene constructs were based on work of Dyson et al[1]. Dashed line indicates cropped membrane to remove empty lane. (b) Coomassie-stained SDS-PAGE (left) and immunoblot analysis (right) of whole cell lysates derived from *E. coli* SHuffle T7 Express *lysY* cells carrying plasmid pET28a(+) encoding each of the indicated constructs. For immunoblots in (a), an equivalent amount of total protein was loaded in each lane. For SDS-PAGE gel and immunoblot in (b), samples were normalized by culture OD_{600} such that an equivalent number of cells were loaded in each lane. Immunoblots in (a) and (b) were probed with anti-polyhistidine antibody (αHis). Control blots were generated by probing membranes with anti-GroEL antibody. Results are representative of three biological replicates. Molecular weight (M_w) markers are shown on left of each blot/gel. Red arrows denote expression products.

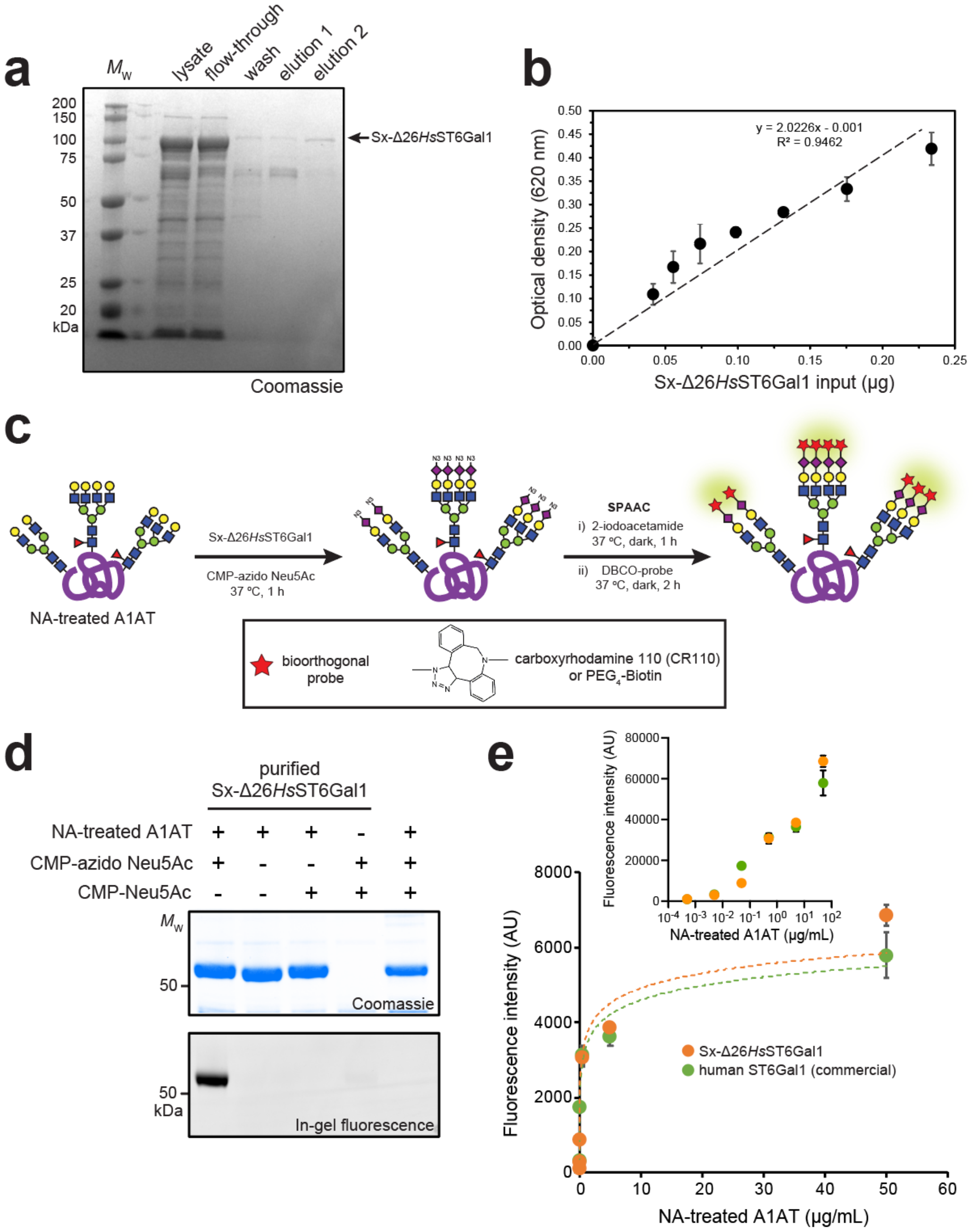

Supplementary Figure 2. Functional characterization of Sx-Δ26*Hs*ST6Gal1. (a) Coomassie-stained SDS-PAGE gel analysis of fractions corresponding to purification of Sx-Δ26*Hs*ST6Gal1 by Ni-NTA chromatography. Gel is representative of three biological replicates. (b) Specific activity of sialyltransferase determined using malachite green phosphate reagents. Absorbance readings (OD_{600}) correspond to amount of released inorganic phosphate from CMP-Neu5Ac after glycosylation. Data are the mean of three biological replicates +/- SEM. (c) Schematic of bioorthogonal click chemistry-based ST assay. Purified human alpha-1 antitrypsin (A1AT) was treated with α2-3,6,8,9 neuraminidase (NA) to remove native sialic acid and used as substrate to evaluate Sx-Δ26*Hs*ST6Gal1-mediated installation of azido-Neu5Ac. Depicted glycans are representative glycoforms of native human A1AT. Azido (N_3-) functional groups on Neu5Ac provide a chemical handle on A1AT for conjugation with carboxyrhodamine 110 (CR110) fluorophore or PEG_4-biotin reporters via strain-promoted azide-alkyne cycloaddition (SPAAC) using dibenzocyclooctyne group (DBCO) as reactive alkyne. (d) Representative SDS-PAGE and immunoblot of reaction products of *in vitro* ST assay. After labeling with CR110, reaction mixtures were separated on SDS-PAGE gel and fluorescence signal of labeled glycoproteins was measured at 501/523 nm $\lambda_{ex}/\lambda_{em}$. Coomassie-stained gel served as loading control. Results are representative of three biological replicates. Molecular weight (M_w) markers are shown at left. (e) Fluorescence corresponding to *in vitro* ST activity of purified Sx-Δ26*Hs*ST6Gal1 and commercial *Hs*ST6Gal1 as function of enzyme concentration. Data are the mean of three biological replicates +/- SEM. Inset is logarithmic representation of same data.

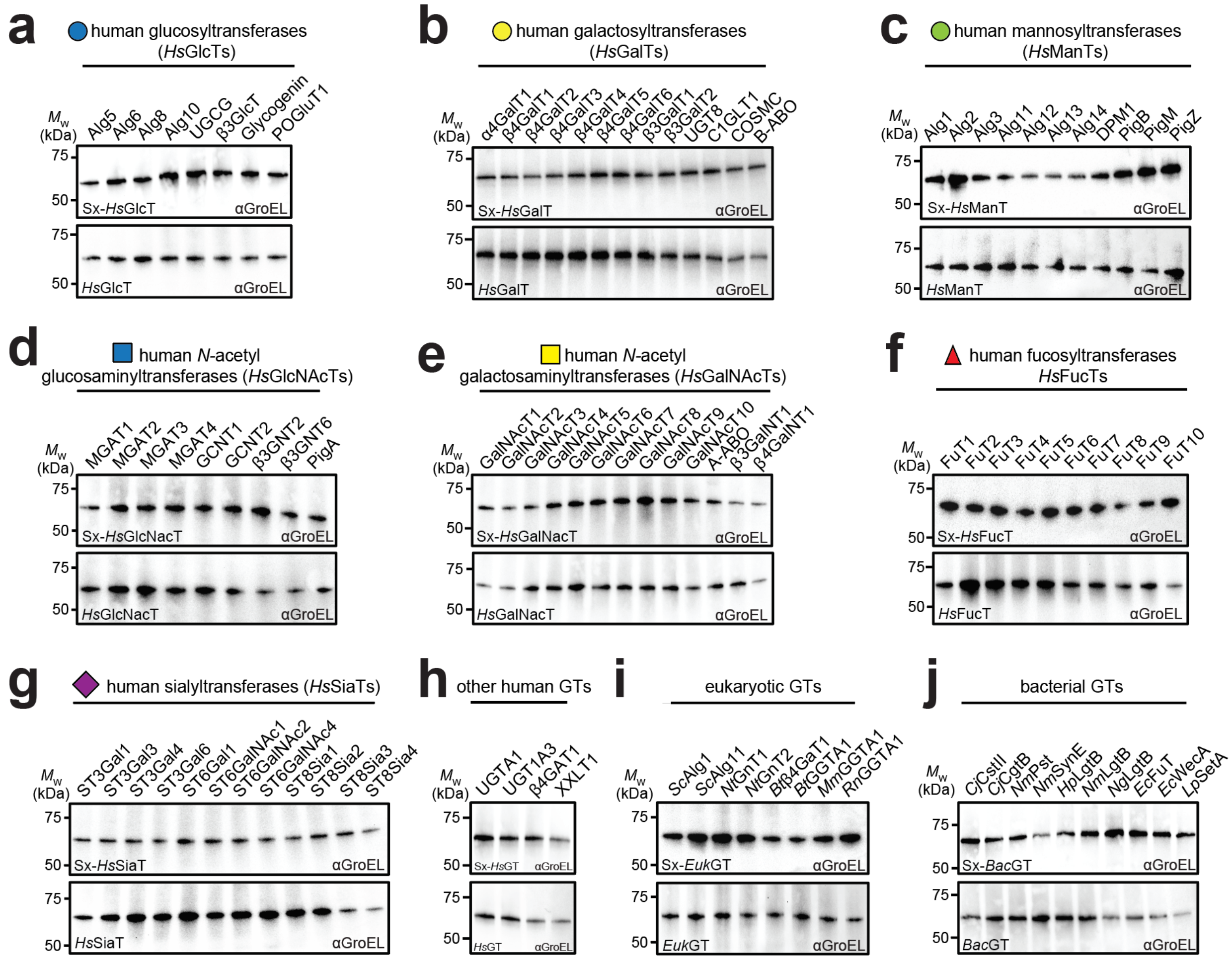

Supplementary Figure 3. Loading control blots for Figure 2 of main manuscript. Control immunoblots corresponding to each panel in main Figure 3 that were generated by loading an identical amount of each sample and probing with anti-GroEL antibody. Immunoblot analysis of soluble fractions derived from either BL21(DE3) or SHuffle T7 Express cells carrying plasmids for Sx-GT (top blot in each panel) or unfused GT (bottom blot in each panel) constructs. GTs were clustered according to origin and activity as follows: (a) human glucosyltransferases (*Hs*GlcTs); (b) human galactosyltransferases (*Hs*GalTs); (c) human mannosyltransferases (*Hs*ManTs); (d) human *N*-acetylglucosaminyltransferases (*Hs*GlcNAcTs); (e) human *N*-acetylgalactosaminyltransferases (*Hs*GalNAcTs); (f) human fucosyltransferases (*Hs*FucTs); (g) human sialyltransferases (*Hs*SiaTs); (h) other human GTs (*Hs*GTs); (i) eukaryotic GTs (*Euk*GTs); and (j) bacterial GTs (*Bac*GTs). Results are representative of three biological replicates. Molecular weight (M_w) markers are indicated at left.

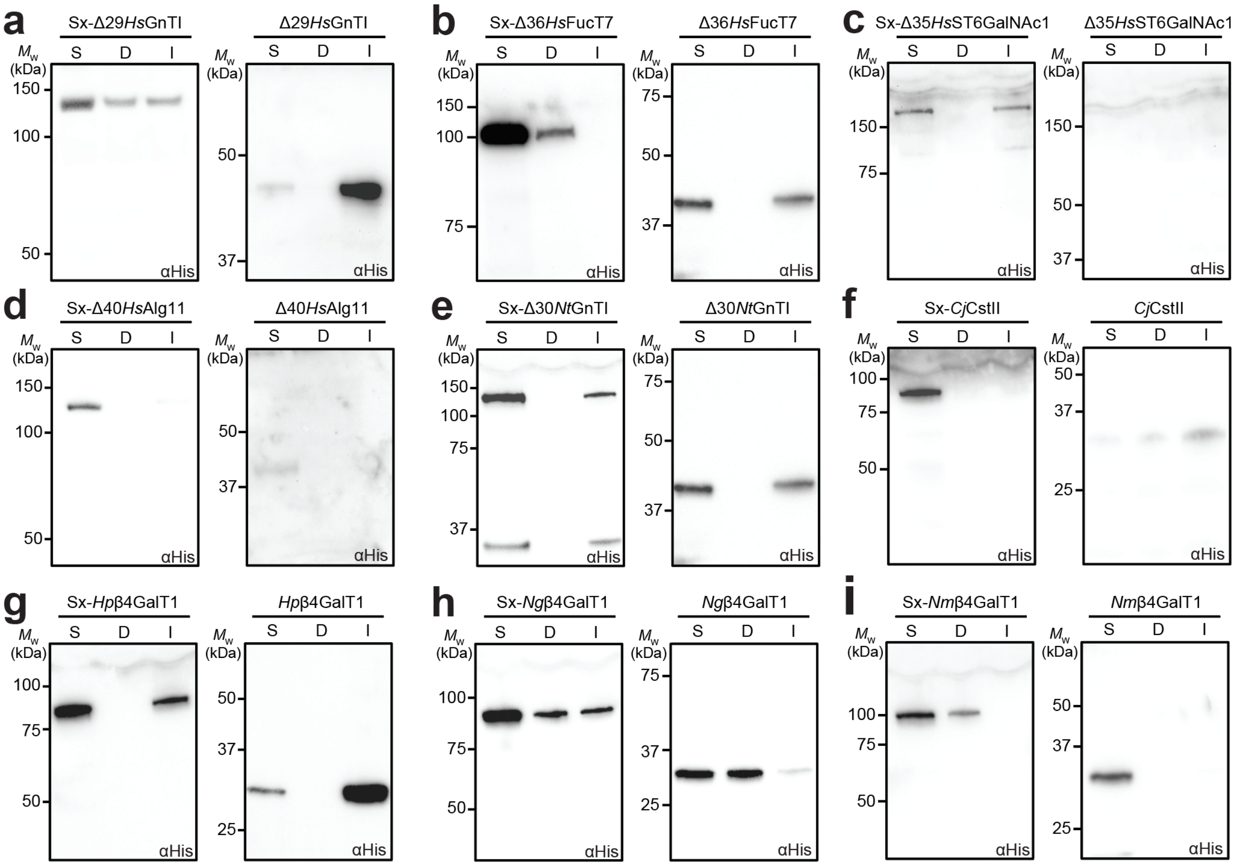

Supplementary Figure 4. Subcellular fractionation analysis of SIMPLEx-reformatted GT expression. Western blot analysis of the soluble (S), detergent-solubilized (D), and insoluble (I) fractions prepared from either BL21(DE3) or SHuffle T7 Express cells carrying plasmid pET28a(+) encoding either Sx-GT (left blot in each panel) or GT (right blot in each panel) constructs corresponding to the following GTs: (a) Δ29*Hs*GnTI; (b) Δ36*Hs*FucT7; (c) Δ35*Hs*ST6 GalNAc1; (d) Δ40*Hs*Alg11; (e) Δ30*Nt*GnTI; (f) *Cj*CsTII: (g) *Hp*β4GalT1; (h) *Ng*β4GalT1; and (i) *Nm*β4GalT1. An equivalent amount of total protein was loaded in each lane. Blots were probed with anti-polyhistidine antibody (αHis). Results are representative of three biological replicates. Molecular weight (M_w) markers are shown on the left.

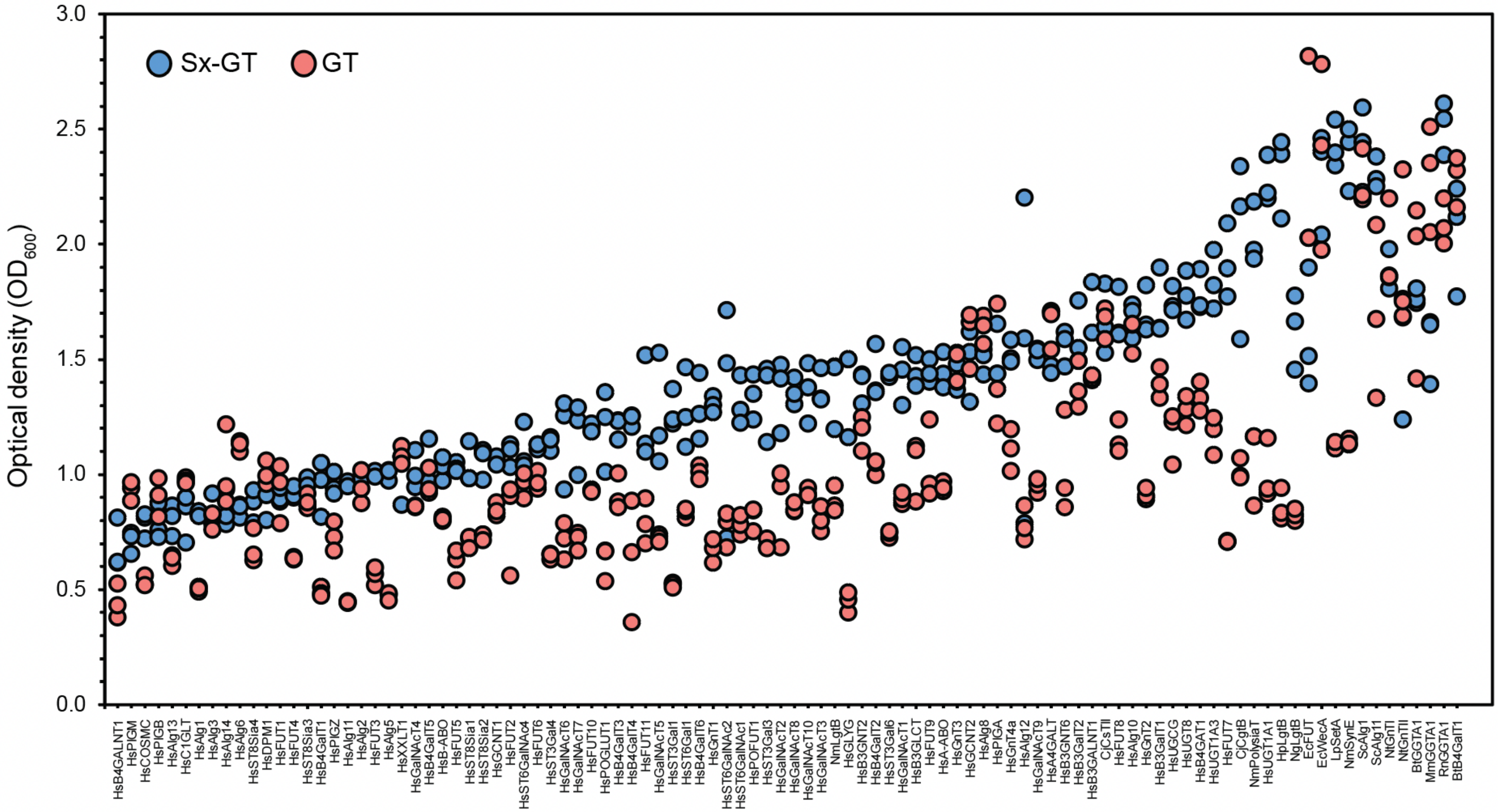

Supplementary Figure 5. Cell density of *E. coli* cultures expressing GT enzymes. Representative data points for cultures expressing SIMPLEx-reformatted GT fusions (Sx-GT; blue) or unfused GTs (red). All data points for each GT were plotted on the same axis. Final cell density values were recorded as OD_{600} readings taken after 16-18 h of growth. Graph depicts three biological replicates for each construct.

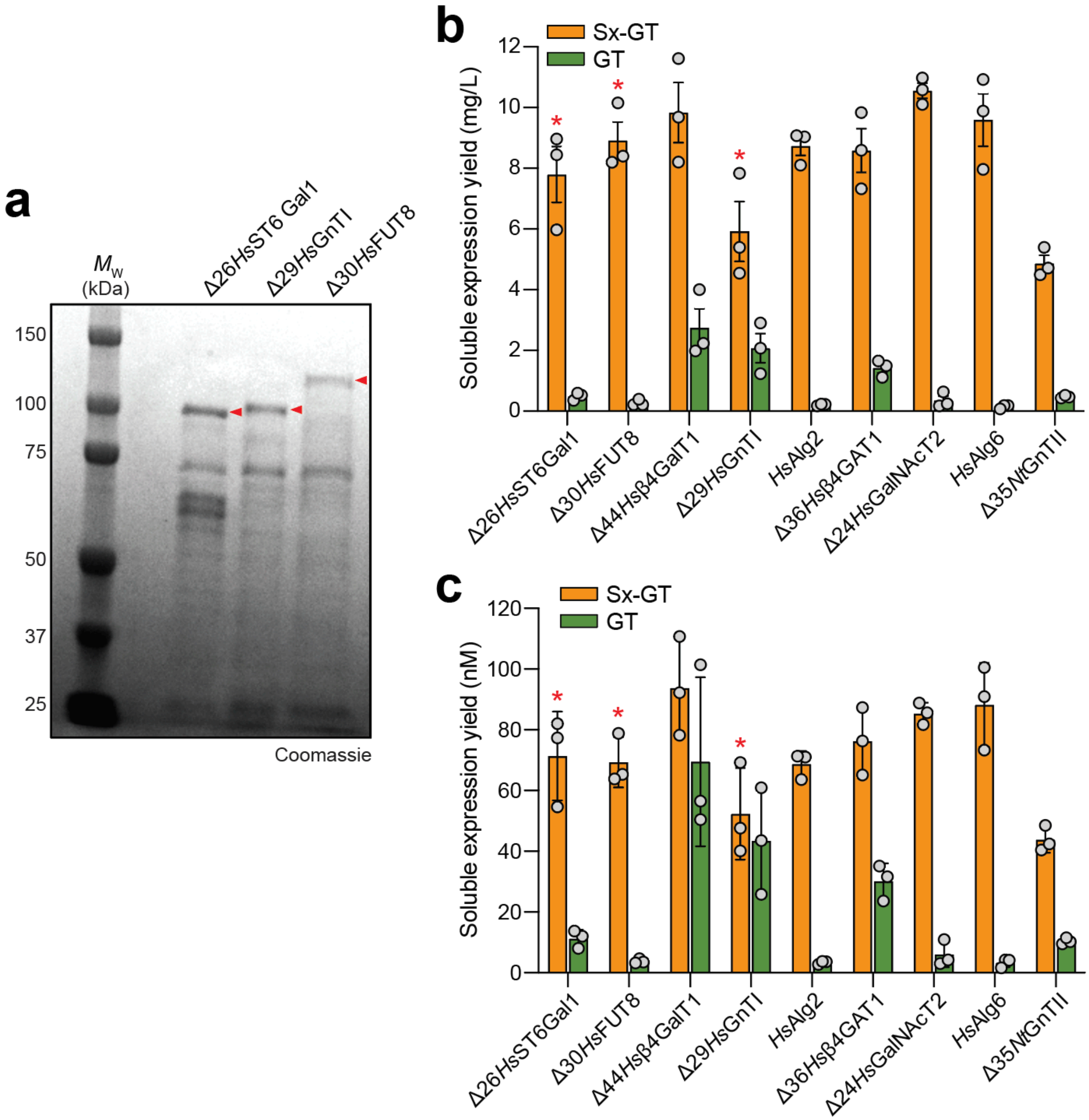

Supplementary Figure 6. Yield quantification for select SIMPLEx-reformatted GTs. (a) Coomassie-stained SDS-PAGE gel analysis of three representative Sx-GTs (Sx-Δ26*hs*ST6Gal1, Sx-Δ29*hs*GnT1, Sx-Δ30*hs*FUT8) following expression and purification by Ni-NTA chromatography. An equivalent amount of total protein was loaded in each lane. Results are representative of three biological replicates. Molecular weight (M_w) marker is shown on the left. Red arrows denote full-length expression products. Soluble protein yields determined on a (b) mass basis (mg/L) and a (c) molar basis (nM). Each indicated protein was purified as in (a) using Ni-NTA chromatography from 1-L cultures of *E. coli* carrying each SIMPLEx-reformatted GT construct as indicated. *E. coli* cultures expressing unfused GT constructs served as controls. Yield values are representative of three biological replicates and error bars represent SEM. Red asterisks denote purified proteins depicted in (a).

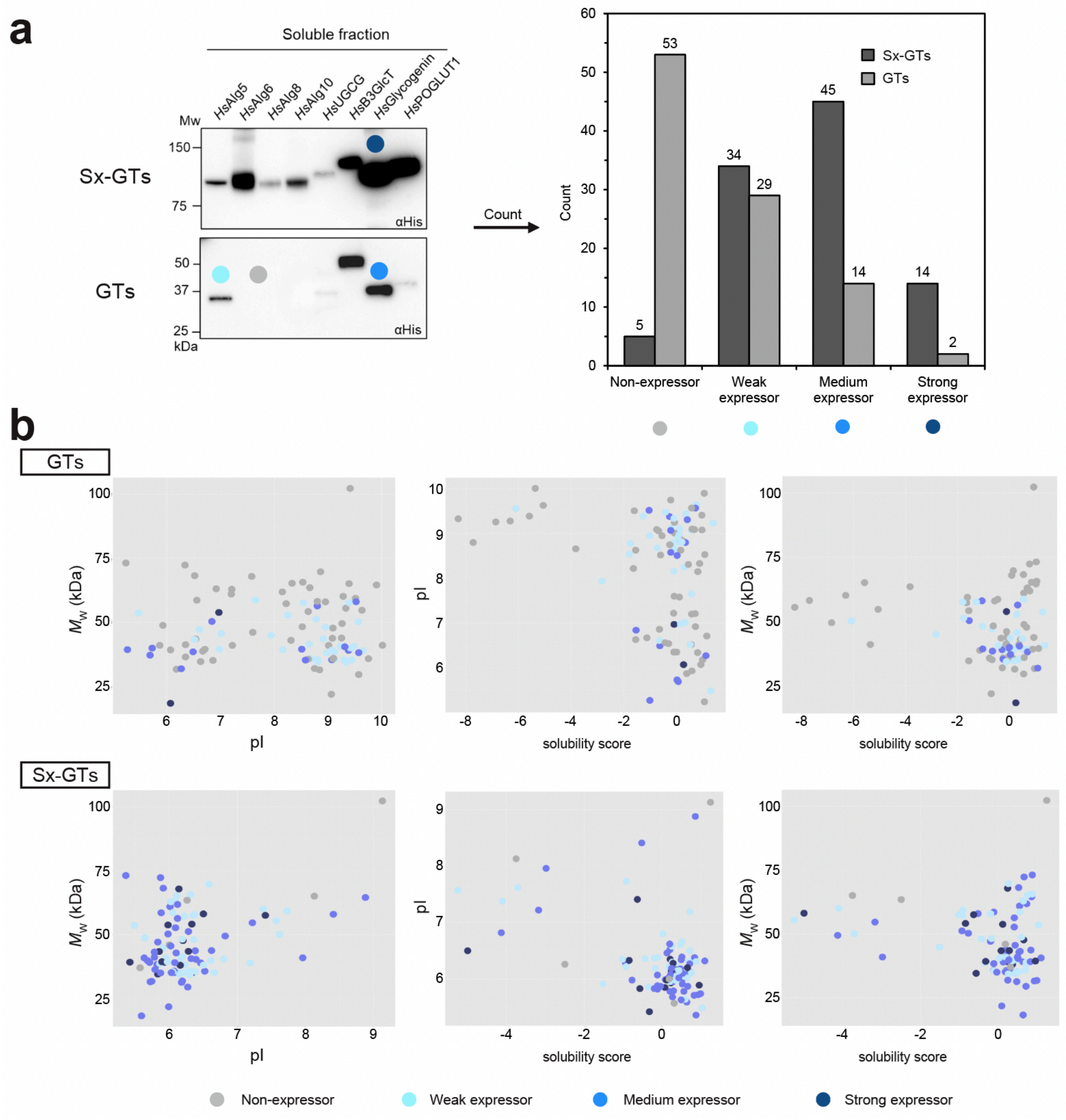

Supplementary Figure 7. Physicochemical properties of GTs that correlate with successful expression. (a) Immunoblot analysis of SIMPLEx-reformatted GTs (Sx-GTs) and unfused GTs (GTs) demonstrate expression score assignment. Blots were probed with anti-polyhistidine antibody (αHis) and results are representative of three biological replicates. Based on relative band intensities from immunoblot analysis, each GT as a SIMPLEx construct or unfused enzyme was categorized as non-expressor (score 0; grey circle), weak expressor (score 1; cyan circle), medium expressor (score 2; light blue circle), and strong expressor (score 3; dark blue circle). Bar graph summarizes total number of GTs for each category, comparing SIMPLEx and unfused formats as indicated. (b) Scatter plots relating the following physicochemical properties: (i) protein molecular weight (M_w) excluding added mass from ΔspMBP and ApoAI* domains; (ii) protein isoelectric point (pI); and (iii) protein solubility score as calculated by Protein-Sol server. Individual data points colored according to their expressor score category. Plots were generated using R version 3.4.2 software. All data used for analysis are provided in Supplementary Dataset 1.

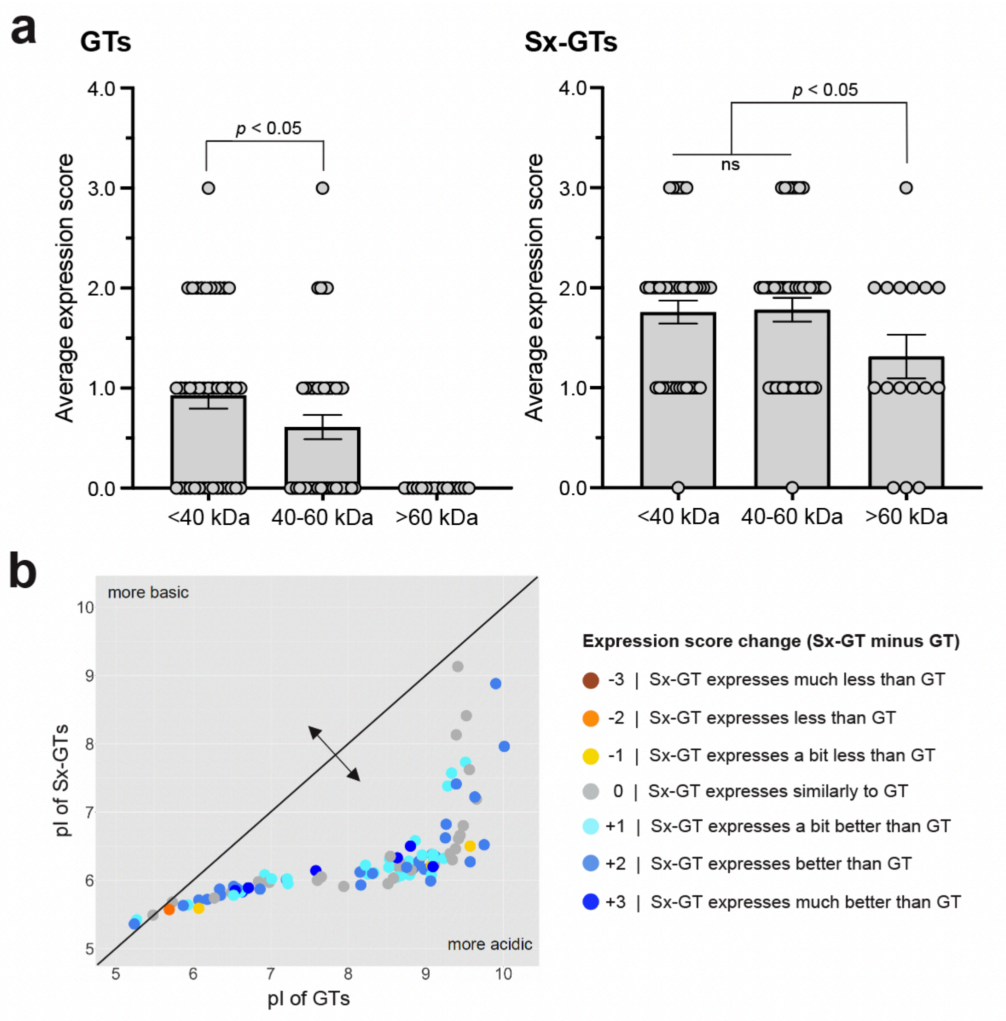

Supplementary Figure 8. Relationship between soluble expression, protein size and isoelectric point. (a) Expression scores for unfused GTs (GTs) or SIMPLEx-reformatted GTs (Sx-GTs) as a function of protein molecular weight to provide average expression score ($\overline{Ex}$) for small (<40 kDa), medium (40-60 kDa), and large (>60 kDa) proteins. Note that the added molecular weight from ΔspMBP and ApoAI* domains of the SIMPLEx construct was excluded from size classification. Graphs depict mean of expression scores determined in Supplementary Fig. 7a +/- SEM. Statistical significance was determined by Welch's two-sided *t*-test ($p < 0.05$ considered significant; ns, not significant), leading to the following *p*-values: 0.0423 for Sx-GTs <40 kDa vs. >60 kDa; 0.0467 for Sx-GTs 40-60 kDa vs. >60 kDa; and 0.0464 for GTs <40 kDa vs. 40-60 kDa. (b) Scatter plot of protein isoelectric point (pI) of unfused and SIMPLEx-fused GTs. Data points were labeled according to change in expression score difference between SIMPLEx-fused and unfused GTs as indicated in legend. Diagonal line represents no change in pI due to SIMPLEx fusion. Arrows signify shift towards a more basic or more acidic protein following SIMPLEx fusion. Plots were generated using R version 3.4.2 software. All data used for analysis are provided in Supplementary Dataset 1.

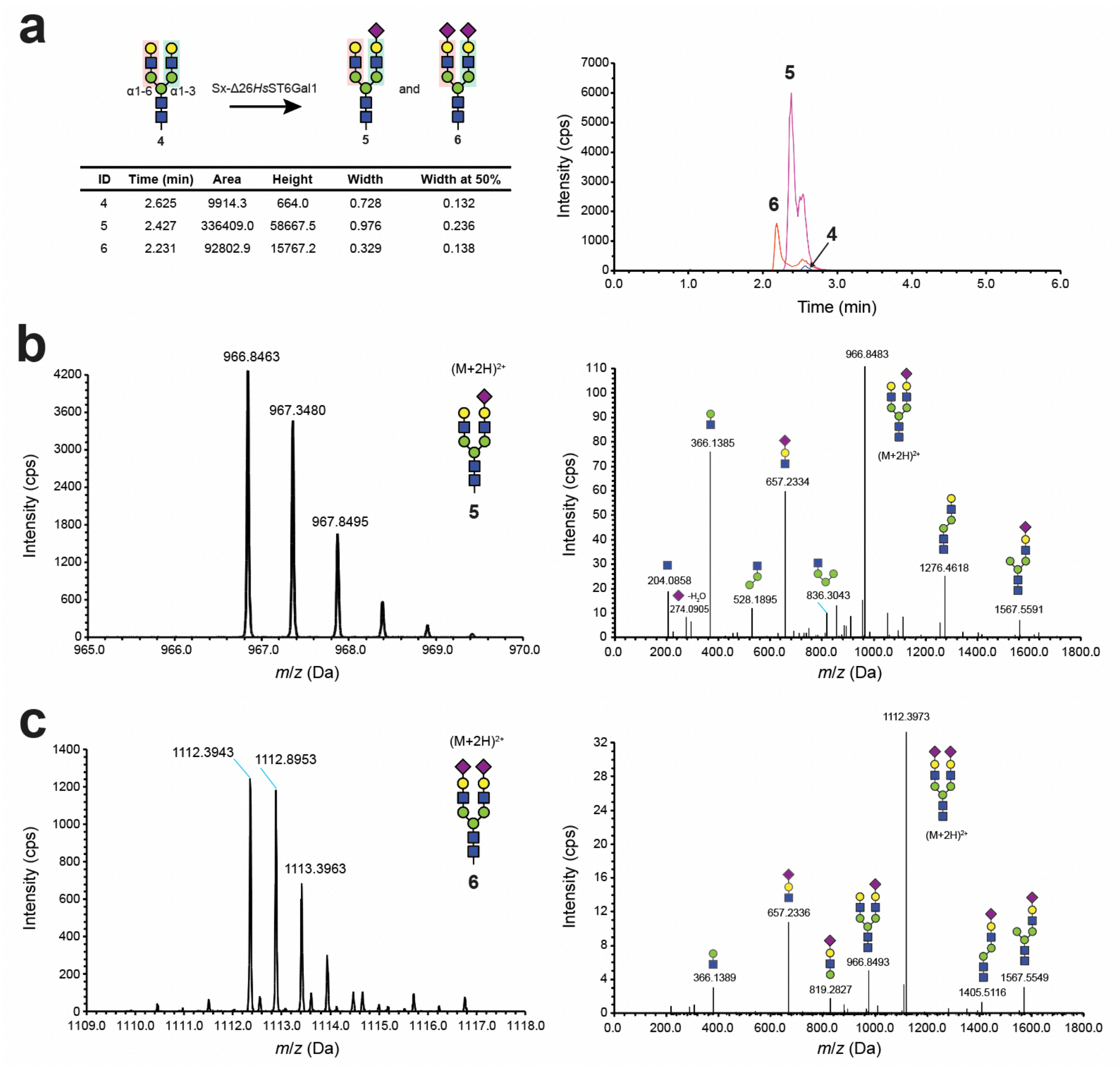

ID	Time (min)	Area	Height	Width	Width at 50%
4	2.625	9914.3	664.0	0.728	0.132
5	2.427	336409.0	58667.5	0.976	0.236
6	2.231	92802.9	15767.2	0.329	0.138

Supplementary Figure 9. MS analysis of sialylated *N*-glycans from cell-free remodeling. (a) HILIC-LC-MS chromatogram of cell-free reaction to install sialic acid on glycan 4 using Sx-Δ26*Hs*ST6Gal1. Schematic for stepwise conversion of glycan 4 to 5 and 6 is shown, with the preferred substrate for *Hs*ST6Gal1, the α1–3Man branch, highlighted in green and the less-preferred α1–6Man branch highlighted in red. (b) MS (left panel) and MS/MS (right panel) spectra of the doubly charged glycan at *m*/*z* 966.8463. Positive ion MS/MS fragmentation pattern confirmed the identity of A2G2S1 product. (c) MS (left panel) and MS/MS (right panel) spectra of the doubly charged glycan at *m*/*z* 1112.3943. Positive ion MS/MS fragmentation pattern confirmed the identity of A2G2S2 product.

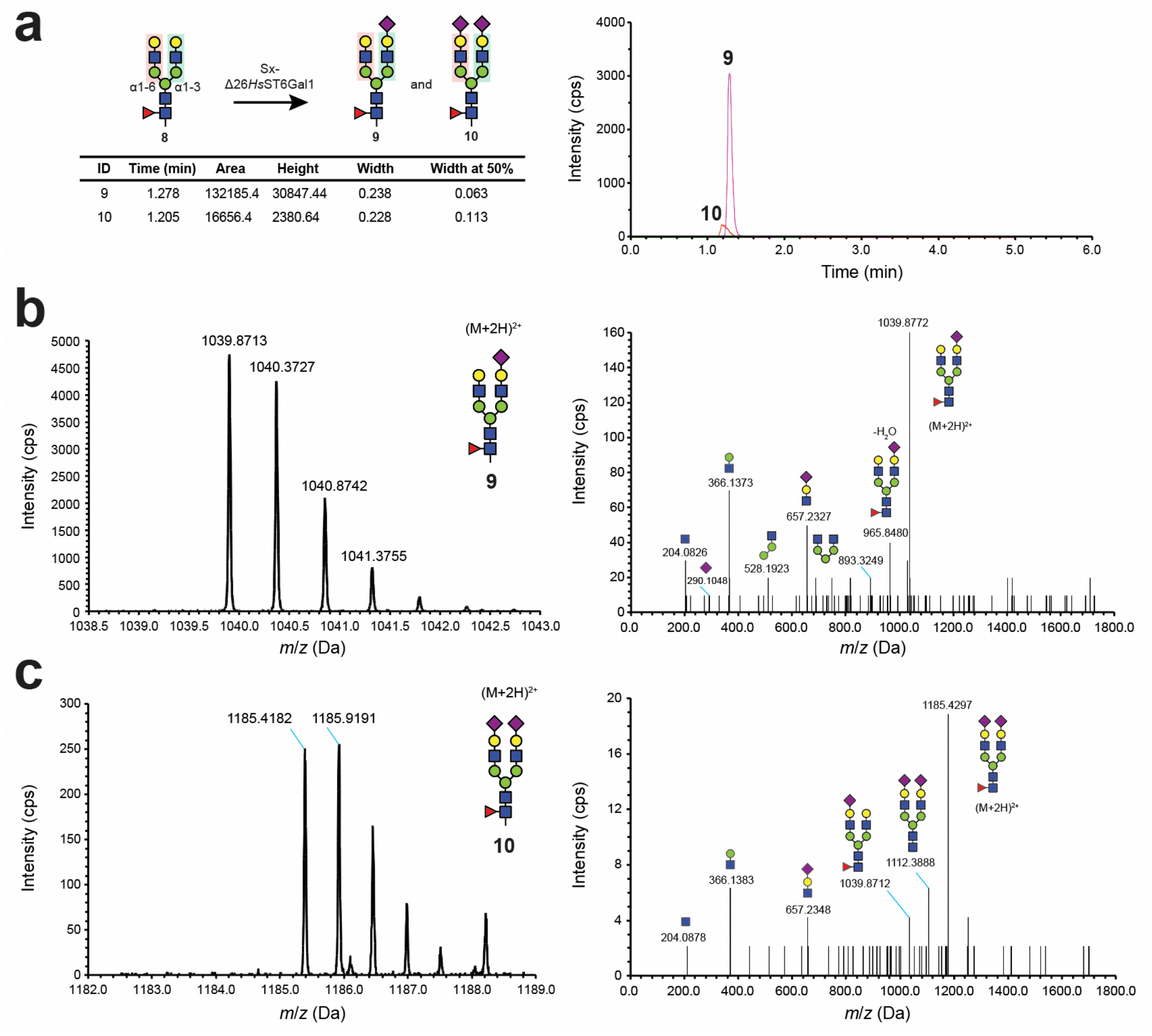

ID	Time (min)	Area	Height	Width	Width at 50%
9	1.278	132185.4	30847.44	0.238	0.063
10	1.205	16656.4	2380.64	0.228	0.113

Supplementary Figure 10. MS analysis of sialylated, core-fucosylated *N*-glycans from cell-free remodeling. (a) HILIC-LC-MS chromatogram of cell-free reaction to install sialic acid on glycan 8 using Sx-Δ26*Hs*ST6Gal1. Schematic for stepwise conversion of glycan 8 to 9 and 10 is shown, with the preferred substrate for *Hs*ST6Gal1, the α1–3Man branch, highlighted in green and the less-preferred α1–6Man branch highlighted in red. (b) MS (left panel) and MS/MS (right panel) spectra of the doubly charged glycan at *m*/*z* 1039.8713. Positive ion MS/MS fragmentation pattern confirmed the identity of A2G2S1 product. (c) MS (left panel) and MS/MS (right panel) spectra of the doubly charged glycan at *m*/*z* 1185.4182. Positive ion MS/MS fragmentation pattern confirmed the identity of A2G2S2 product.

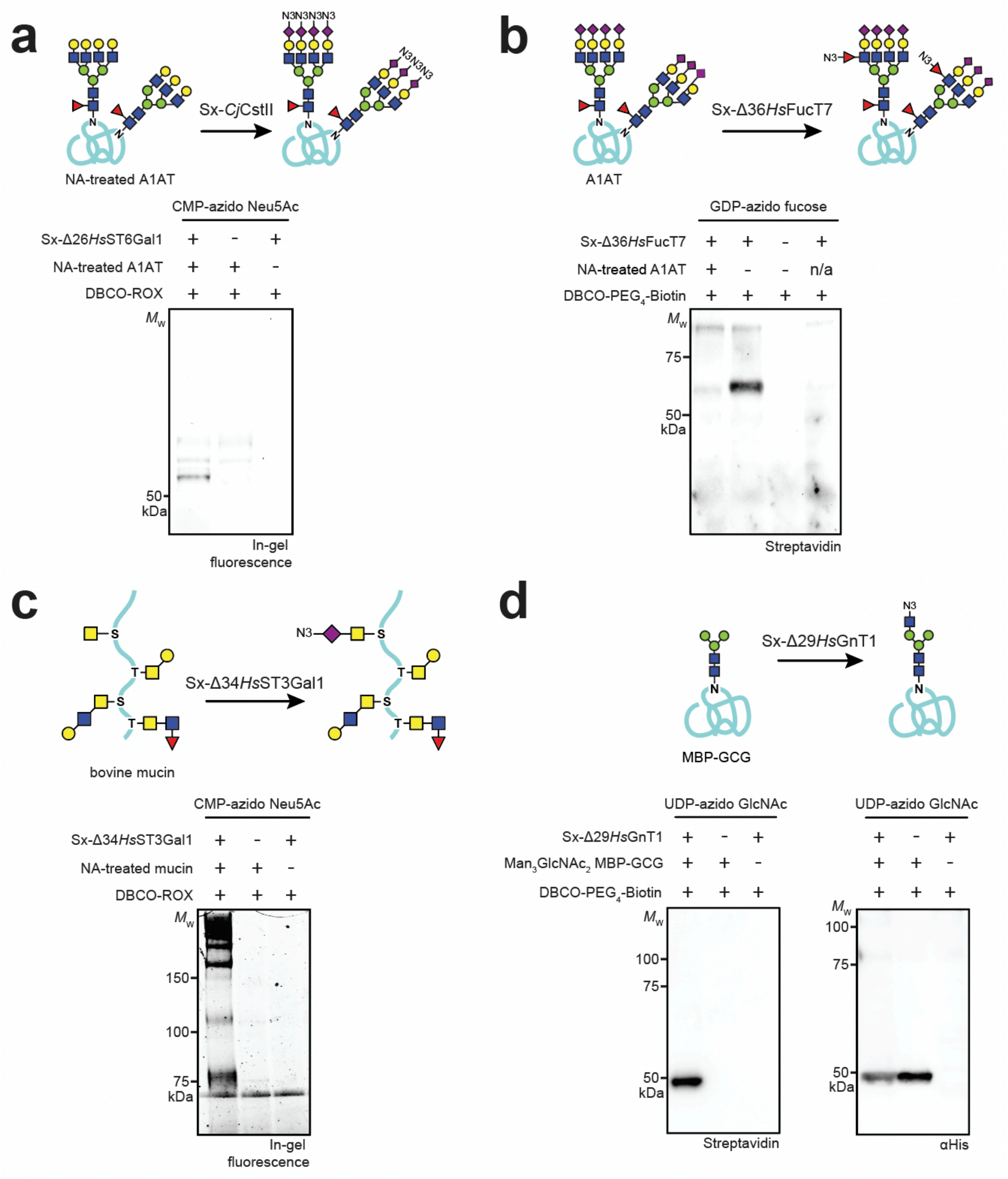

Supplementary Figure 11. Remodeling glycans on therapeutic glycoproteins with Sx-GTs. Cell-free reactions catalyzing: (a) sialyltransferase reaction on *N*-glycan of neuraminidase (NA)-treated A1A1 using Sx-*Cj*CstII; (b) fucosyltransferase reaction on *N*-glycan of NA-treated A1AT using Sx-Δ36*Hs*FucT7; (c) sialyltransferase reaction on *O*-glycan of NA-treated bovine submaxillary mucin using Sx-Δ34*Hs*ST3Gal1; and (d) *N*-acetylglucosaminyl transferase reaction on $Man_3GlcNAc_2$ *N*-glycan of MBP-glucagon fusion protein[2] using Sx-Δ29*Hs*GnT1. In all cases, reactions were performed using nucleotide-activated sugars modified with azide group as indicated. Following Sx-GT-catalyzed reactions, glycoprotein products were labeled with either dibenzocyclooctyne-carboxyrhodamine 110 (DBCO-CR110) or DBCO-PEG_4-biotin reporters via SPACC reaction. In-gel fluorescence analysis was used to detect glycoproteins modified with fluorescent CR110 reporter while immunoblot analysis using streptavidin-HRP was used to detect glycoproteins modified with biotin reporter. Results are representative of three biological replicates. Molecular weight (M_w) markers are shown at left.

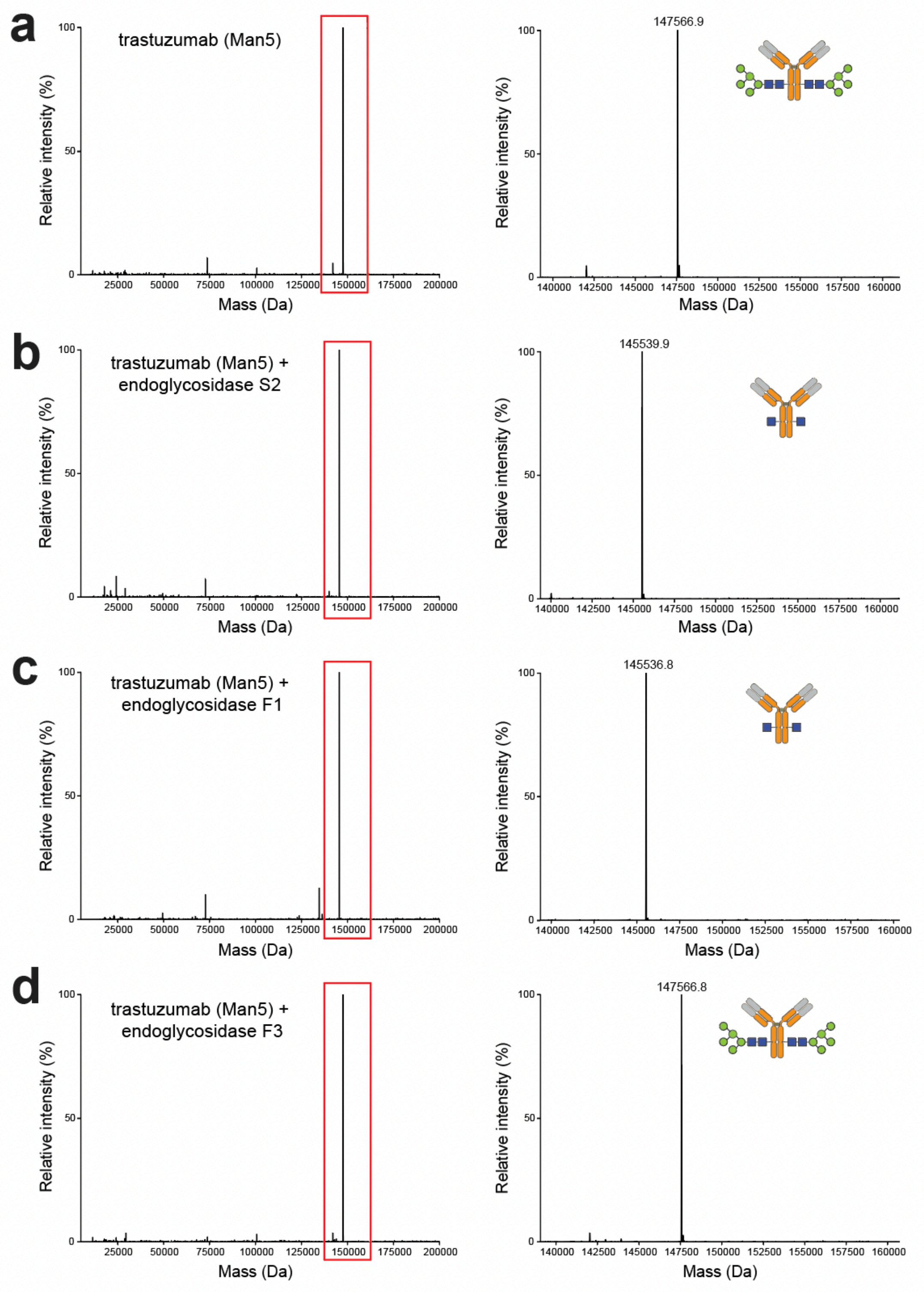

Supplementary Figure 12. Glycosidase sensitivity of *N*-glycans on trastuzumab. Deconvoluted MS analysis of intact trastuzumab derived from Expi293F™ GnTI⁻ cells following incubation with: (a) PBS; (b) endoglycosidase S2; (c) endoglycosidase F1; and (d) endoglycosidase F3. All reactions were carried out at 37 ºC for 16 h in 10-µL reaction volume. Full spectra in the range of 25-200 kDa are shown in the panels on the left. Red box indicates region between 140-160 kDa and the spectra for this mass range is provided in the panels on the right. Structures of anticipated *N*-glycoprotein products are provided within each spectrum.

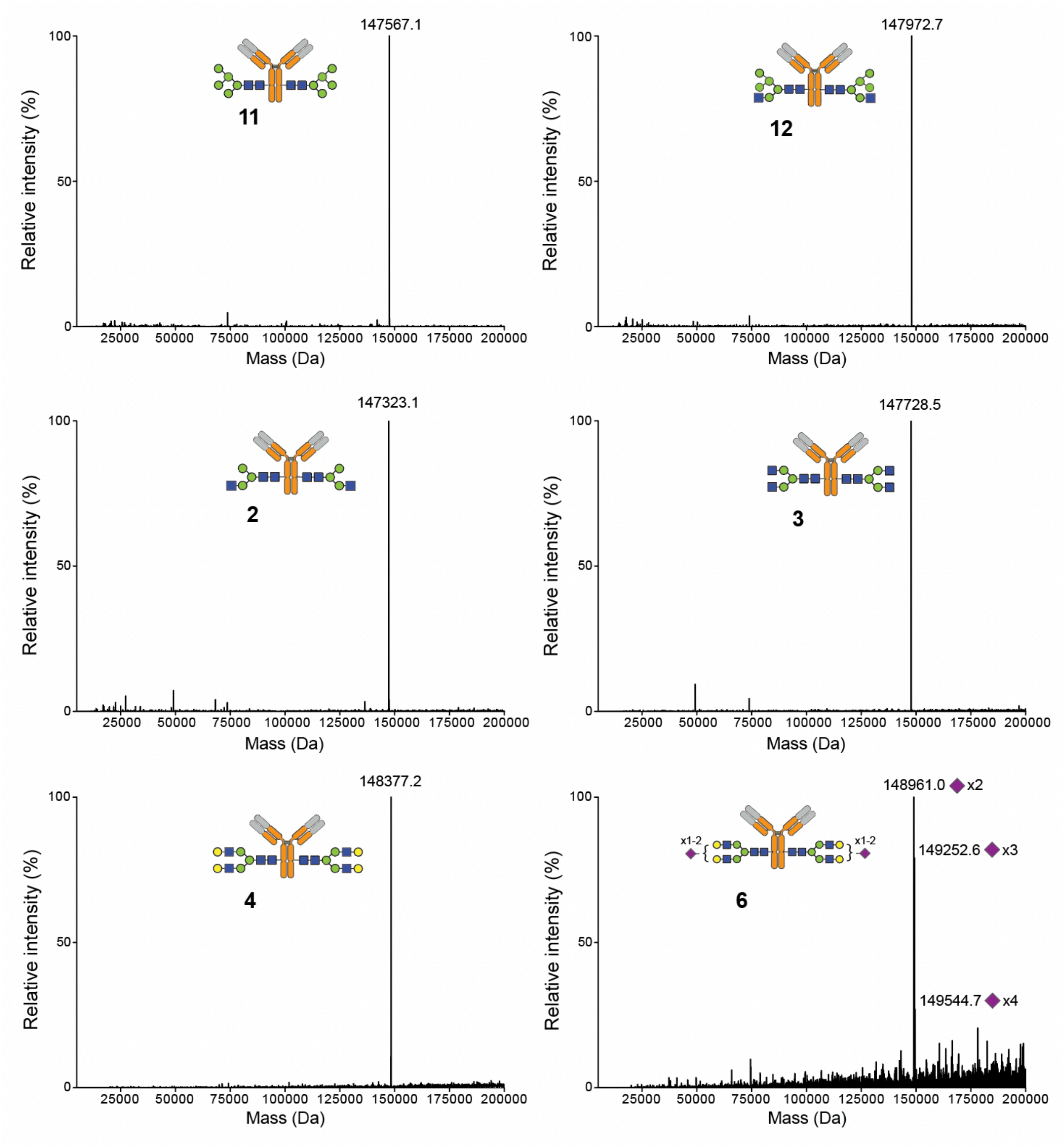

Supplementary Figure 13. MS spectra for trastuzumab glycoforms. Full MS spectra in the range of 0-200 kDa corresponding to each glycoform of trastuzumab detected in Figure 5 of the main manuscript. Structures of anticipated *N*-linked glycoprotein products are provided in each spectrum.

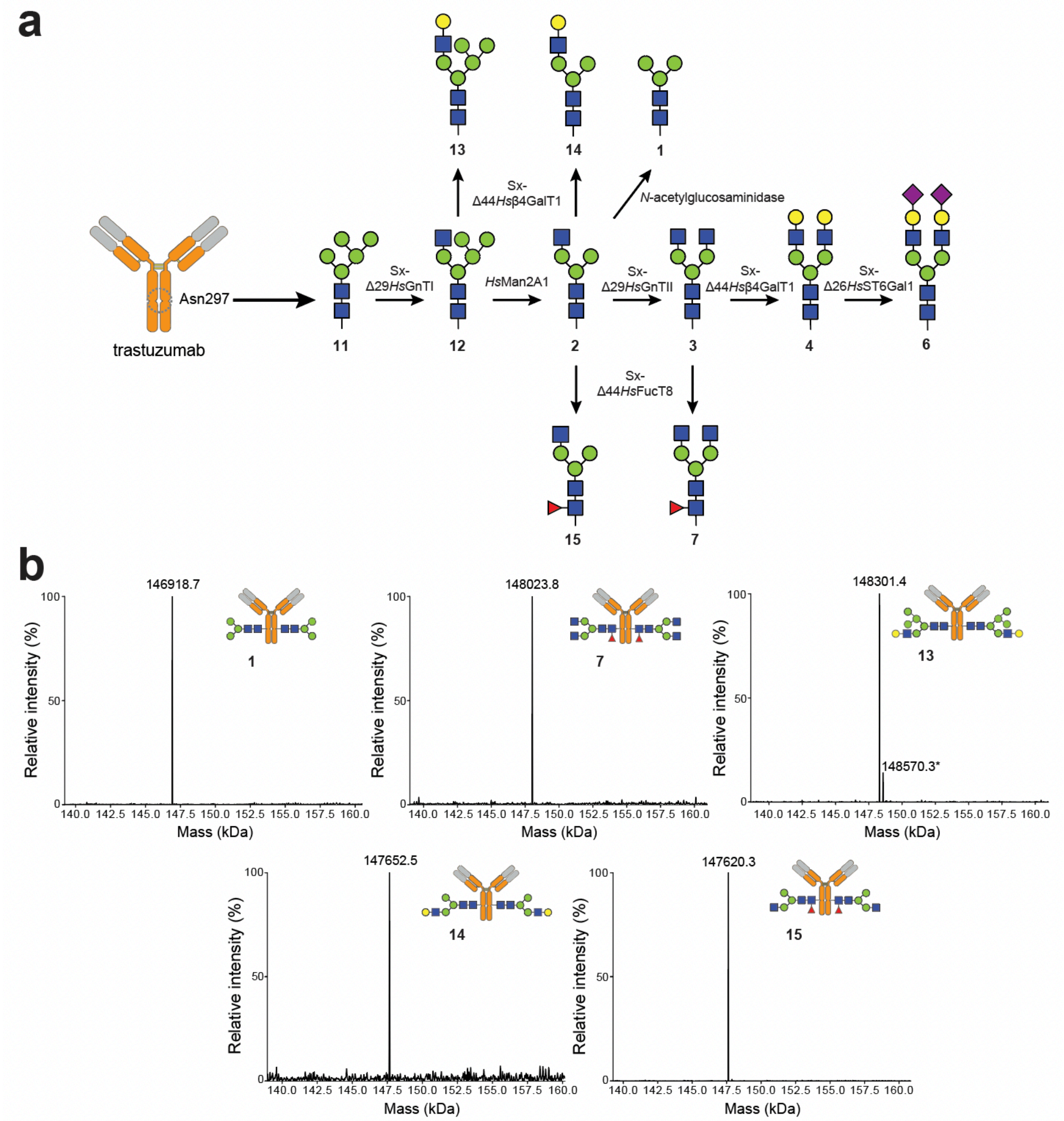

Supplementary Figure 14. Remodeling of IgG-Fc *N*-glycans on trastuzumab using Sx-GTs. (a) Extended schematic of bioenzymatic routes to hybrid- and complex-type *N*-glycan structures linked to N297 of trastuzumab. Trastuzumab bearing $Man_5GlcNAc_2$ glycan (M5; glycan 11) derived from glycoengineered HEK293F lacking GnTI activity was used as a glycan primer. Subsequent cell-free glycan remodeling reactions yielded the following *N*-glycan structures: 1 (M3); 2 (G0-GlcNAc); 3 (G0); 4 (G2); 6 (G2S2); 7(G0F); 12 (M5+GlcNAc); 13 (M5+GlcNAcGal); 14 (M3+GlcNAc); and 15 (G0F-GlcNAc). For complete glycan list with chemical structures, see Supplementary Table 2. SIMPLEx-reformatted GTs and glycosidases for each synthesis step are provided above reaction arrow. (b) Deconvoluted LC-MS spectra in 140–160 kDa range using intact antibody analysis of enzymatically-derived product glycans 1, 7, and 13-15. Structures of the anticipated *N*-glycoprotein products are provided in each spectrum. Asterisk indicates unidentified product.

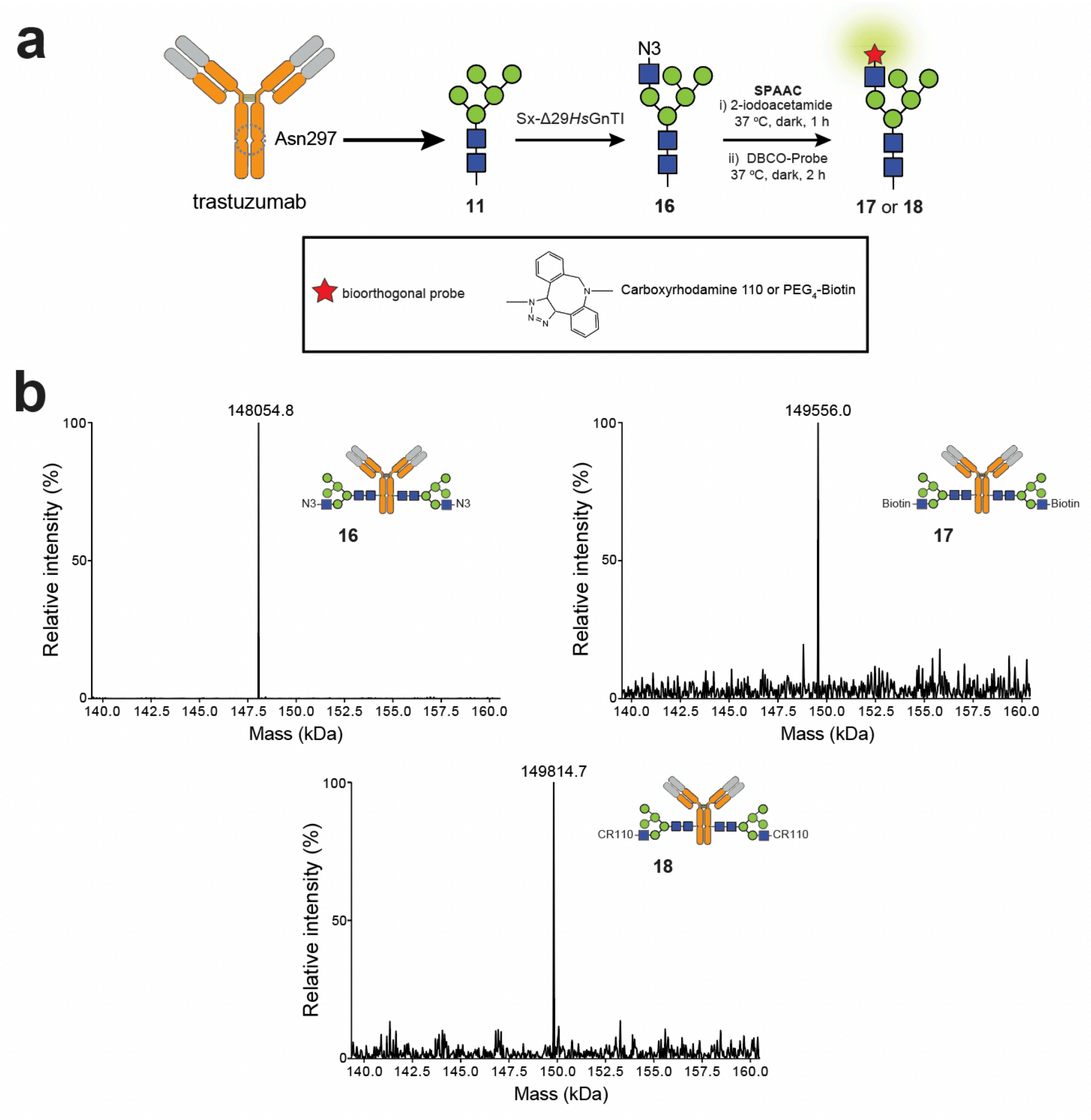

Supplementary Figure 15. Remodeling of IgG-Fc *N*-glycans on trastuzumab using Sx-GTs. (a) Schematic of bioenzymatic routes to unnatural *N*-glycan structures linked to N297 of trastuzumab. Trastuzumab bearing $Man_5GlcNAc_2$ glycan (M5; glycan 11) was used as a glycan primer to generate *N*-glycan 16 (M5-GlcNAz). Subsequent strain-promoted cycloaddition reaction was performed to install either DBCO-PEG_4-Biotin or DBCO-carboxyrhodamine (CR110) to the azide group, yielding glycan 17 and glycan 18, respectively. (b) Deconvoluted LC-MS spectra in 140–160 kDa range using intact antibody analysis of enzymatically-derived product glycans 16–18. Structures of the anticipated *N*-glycoprotein products are provided in each spectrum.

Supplementary Table 1. Strains, cell lines, and plasmids used in this study.

Strain	Genotype	Source
**Bacterial strain**		
DH5α	F$^-$ (Φ80Δ*lacZ*ΔM15,) Δ(*lacIZYA-argF*)U169 *recA1 endA1 hsdR17*(r$_K^-$,m$_K^+$) *phoA supE44* λ$^-$ *thi-1 gyrA96 relA1*	Lab stock
BL21(DE3)	F$^-$ *ompT gal dcm lon hsdSB*(r$_B^-$ m$_B^-$) λ(DE3)	Lab stock
SHuffle® T7 Express *lysY*	MiniF *lysY* (CamR) / *fhuA2 lacZ*::T7 gene1 [*lon*] *ompT ahpC gal* λatt::pNEB3-r1-cDsbC (SpecR, *lacI*^{q}) Δ*trxB sulA11* R(*mcr-73*::miniTn10–TetS)2 [*dcm*] R(*zgb-210*::Tn10–TetS) *endA1* Δ*gor* Δ(*mcrC-mrr*)114::IS10	NEB
Origami2(DE3) *gmd*::kan Δ*waaL*	*E. coli* Origami2 cells carrying marked deletion of *gmd* gene and unmarked deletion of *waaL* gene	Reference [3]
**Yeast strain**		
SBY49	MATa *pep4*Δ ::LEU2 *prb1*Δ ::LEU2 *ura3-52 his3-200 leu2-3*,*112 lys2-801 suc2-9*	Scott Emr lab
**Cell line**		
HEK293T		ATCC
FreeStyle™ 293-F		Thermo Fisher
Expi293F™ GnTI-		Thermo Fisher

**Plasmid**	**Description**	**Source**
pET28a(+)	T7 *lac* promoter; Kan^R	Novagen
pET28a-SIMPLEx	MCS flanked by N-terminal *E. coli* MBP lacking its signal peptide (Δ1-26; ΔspMBP) and C-terminal truncated human ApoAI (Δ1-43; ApoAI*) followed by 6xHis tag cloned in pET28a(+); Kan^R	Reference [4]
pET28a-SIMPLEx-GFP	superfolder GFP (sfGFP) cloned in MCS of pET28a-SIMPLEx; Kan^R	Reference [4]
pJL1-sfGFP	T7 *lac* promoter, cell-free expression vector encoding superfolder GFP (sfGFP) gene; Kan^R	Reference [5]
pVITRO1-Trastuzumab-IgG1/κ	EM7 promoter, encoding HER2/neu receptor specific humanized IgG1/κ antibody isotype; Hygro^R	Reference [6]
pcDNA3	CMV promoter; Amp^R	Laboratory stock
pYS338	CPS promoter, galactose inducible vector; Amp^R	Scott Emr lab
pET28a-SIMPLEx-Δ25*Hs*FUT1	truncated human FUT1 (Δ1-25) cloned in MCS of pET28a-SIMPLEx; Kan^R	This study
pET28a-SIMPLEx-Δ28*Hs*FUT2	truncated human FUT2 (Δ1-28) cloned in MCS of pET28a-SIMPLEx; Kan^R	This study
pET28a-SIMPLEx-Δ34*Hs*FUT3	truncated human FUT3 (Δ1-34) cloned in MCS of pET28a-SIMPLEx; Kan^R	This study
pET28a-SIMPLEx-Δ172*Hs*FUT4	truncated human FUT4 (Δ1-172) cloned in MCS of pET28a-SIMPLEx; Kan^R	This study
pET28a-SIMPLEx-Δ34*Hs*FUT5	truncated human FUT5 (Δ1-34) cloned in MCS of pET28a-SIMPLEx; Kan^R	This study
pET28a-SIMPLEx-Δ34*Hs*FUT6	truncated human FUT6 (Δ1-34) cloned in MCS of pET28a-SIMPLEx; Kan^R	This study
pET28a-SIMPLEx-Δ36*Hs*FUT7	truncated human FUT7 (Δ1-36) cloned in MCS of pET28a-SIMPLEx; Kan^R	This study
pET28a-SIMPLEx-Δ30*Hs*FUT8	truncated human FUT8 (Δ1-30) cloned in MCS of pET28a-SIMPLEx; Kan^R	This study
pET28a-SIMPLEx-Δ32*Hs*FUT9	truncated human FUT9 (Δ1-32) cloned in MCS of pET28a-SIMPLEx; Kan^R	This study
pET28a-SIMPLEx-Δ31*Hs*FUT10	truncated human FUT10 (Δ1-31) cloned in MCS of pET28a-SIMPLEx; Kan^R	This study
pET28a-SIMPLEx-Δ24*Hs*FUT11	truncated human FUT11 (Δ1-24) cloned in MCS of pET28a-SIMPLEx; Kan^R	This study
pET28a-SIMPLEx-Δ26*Hs*POFUT1	truncated human POFUT1 (Δ1-26) cloned in MCS of pET28a-SIMPLEx; Kan^R	This study
pET28a-SIMPLEx-Δ34*Hs*ST3Gal1	truncated human ST3Gal1 (Δ1-34) cloned in MCS of pET28a-SIMPLEx; Kan^R	This study
pET28a-SIMPLEx-Δ28*Hs*ST3Gal3	truncated human ST3Gal3 (Δ1-28) cloned in MCS of pET28a-SIMPLEx; Kan^R	This study
pET28a-SIMPLEx-Δ26*Hs*ST3Gal4	truncated human ST3Gal4 (Δ1-26) cloned in MCS of pET28a-SIMPLEx; Kan^R	This study
pET28a-SIMPLEx-Δ25*Hs*ST3Gal6	truncated human ST3Gal6 (Δ1-25) cloned in MCS of pET28a-SIMPLEx; Kan^R	This study
pET28a-SIMPLEx-Δ26*Hs*ST6Gal1	truncated human ST6Gal1 (Δ1-26) cloned in MCS of pET28a-SIMPLEx; Kan^R	This study
pET28a-SIMPLEx-*Hs*ST6Gal1	human ST6Gal1 cloned in MCS of pET28a-SIMPLEx; Kan^R	This study

pET28a-SIMPLEx-Δ35*Hs*ST6GalNAc1	truncated human ST3GalNAc1 (Δ1-35) cloned in MCS of pET28a-SIMPLEx; Kan^R	This study
pET28a-SIMPLEx-Δ28*Hs*ST6GalNAc2	truncated human ST3GalNAc2 (Δ1-28) cloned in MCS of pET28a-SIMPLEx; Kan^R	This study
pET28a-SIMPLEx-Δ27*Hs*ST6GalNAc4	truncated human ST3GalNAc4 (Δ1-27) cloned in MCS of pET28a-SIMPLEx; Kan^R	This study
pET28a-SIMPLEx-Δ48*Hs*ST8Sia1	truncated human ST8Sia1 (Δ1-48) cloned in MCS of pET28a-SIMPLEx; Kan^R	This study
pET28a-SIMPLEx-Δ23*Hs*ST8Sia2	truncated human ST8Sia2 (Δ1-23) cloned in MCS of pET28a-SIMPLEx; Kan^R	This study
pET28a-SIMPLEx-Δ33*Hs*ST8Sia3	truncated human ST8Sia3 (Δ1-33) cloned in MCS of pET28a-SIMPLEx; Kan^R	This study
pET28a-SIMPLEx-Δ20*Hs*ST8Sia4	truncated human ST8Sia4 (Δ1-20) cloned in MCS of pET28a-SIMPLEx; Kan^R	This study
pET28a-SIMPLEx-Δ28*Hs*ppGalNAcT1	truncated human ppGalNAcT1 (Δ1-28) cloned in MCS of pET28a-SIMPLEx; Kan^R	This study
pET28a-SIMPLEx-Δ24*Hs*ppGalNAcT2	truncated human ppGalNAcT2 (Δ1-24) cloned in MCS of pET28a-SIMPLEx; Kan^R	This study
pET28a-SIMPLEx-Δ37*Hs*ppGalNAcT3	truncated human ppGalNAcT3 (Δ1-37) cloned in MCS of pET28a-SIMPLEx; Kan^R	This study
pET28a-SIMPLEx-Δ35*Hs*ppGalNAcT4	truncated human ppGalNAcT4 (Δ1-35) cloned in MCS of pET28a-SIMPLEx; Kan^R	This study
pET28a-SIMPLEx-Δ35*Hs*ppGalNAcT5	truncated human ppGalNAcT5 (Δ1-35) cloned in MCS of pET28a-SIMPLEx; Kan^R	This study
pET28a-SIMPLEx-Δ28*Hs*ppGalNAcT6	truncated human ppGalNAcT6 (Δ1-28) cloned in MCS of pET28a-SIMPLEx; Kan^R	This study
pET28a-SIMPLEx-Δ29*Hs*ppGalNAcT7	truncated human ppGalNAcT7 (Δ1-29) cloned in MCS of pET28a-SIMPLEx; Kan^R	This study
pET28a-SIMPLEx-Δ29*Hs*ppGalNAcT8	truncated human ppGalNAcT8 (Δ1-29) cloned in MCS of pET28a-SIMPLEx; Kan^R	This study
pET28a-SIMPLEx-Δ28*Hs*ppGalNAcT9	truncated human ppGalNAcT9 (Δ1-28) cloned in MCS of pET28a-SIMPLEx; Kan^R	This study
pET28a-SIMPLEx-Δ31*Hs*ppGalNAcT10	truncated human ppGalNAcT10 (Δ1-31) cloned in MCS of pET28a-SIMPLEx; Kan^R	This study
pET28a-SIMPLEx-Δ43*Hs*B3GALNT1	truncated human B3GALNT1 (Δ1-43) cloned in MCS of pET28a-SIMPLEx; Kan^R	This study
pET28a-SIMPLEx-Δ25*Hs*B4GALNT1	truncated human B4GALNT1 (Δ1-25) cloned in MCS of pET28a-SIMPLEx; Kan^R	This study
pET28a-SIMPLEx-Δ53*Hs*-A-group	truncated human A-ABO (Δ1-53) cloned in MCS of pET28a-SIMPLEx; Kan^R	This study
pET28a-SIMPLEx-Δ43*Hs*A4GalT	truncated human A4GalT (Δ1-43) cloned in MCS of pET28a-SIMPLEx; Kan^R	This study
pET28a-SIMPLEx-Δ26*Hs*B3GalT1	truncated human B3GalT1 (Δ1-26) cloned in MCS of pET28a-SIMPLEx; Kan^R	This study
pET28a-SIMPLEx-Δ45*Hs*B3GalT2	truncated human B3GalT2 (Δ1-45) cloned in MCS of pET28a-SIMPLEx; Kan^R	This study
pET28a-SIMPLEx-Δ44*Hs*B4GalT1	truncated human B4GalT1 (Δ1-44) cloned in MCS of pET28a-SIMPLEx; Kan^R	This study
pET28a-SIMPLEx-Δ36*Hs*B4GalT2	truncated human B4GalT2 (Δ1-36) cloned in MCS of pET28a-SIMPLEx; Kan^R	This study
pET28a-SIMPLEx-Δ31*Hs*B4GalT3	truncated human B4GalT3 (Δ1-31) cloned in MCS of pET28a-SIMPLEx; Kan^R	This study
pET28a-SIMPLEx-Δ38*Hs*B4GalT4	truncated human B4GalT4 (Δ1-38) cloned in MCS of pET28a-SIMPLEx; Kan^R	This study
pET28a-SIMPLEx-Δ35*Hs*B4GalT5	truncated human B4GalT5 (Δ1-35) cloned in MCS of pET28a-SIMPLEx; Kan^R	This study
pET28a-SIMPLEx-Δ35*Hs*B4GalT6	truncated human B4GalT6 (Δ1-35) cloned in MCS of pET28a-SIMPLEx; Kan^R	This study
pET28a-SIMPLEx-Δ53*Hs*-B-group	truncated human B-ABO (Δ1-53) cloned in MCS of pET28a-SIMPLEx; Kan^R	This study
pET28a-SIMPLEx-Δ20*Hs*UGT8	human UGT8 cloned in MCS of pET28a-SIMPLEx; Kan^R	This study
pET28a-SIMPLEx-Δ29*Hs*C1GLT	human C1GLT cloned in MCS of pET28a-SIMPLEx; Kan^R	This study
pET28a-SIMPLEx-*Hs*COSMC	human COSMC cloned in MCS of pET28a-SIMPLEx; Kan^R	This study
pET28a-SIMPLExΔ23 *Hs*Alg1	truncated human Alg1 (Δ1-23) cloned in MCS of pET28a-SIMPLEx; Kan^R	This study
pET28a-SIMPLEx-*Hs*Alg2	human Alg2 cloned in MCS of pET28a-SIMPLEx; Kan^R	This study
pET28a-SIMPLEx-*Hs*Alg3	human Alg3 cloned in MCS of pET28a-SIMPLEx; Kan^R	This study
pET28a-SIMPLEx-Δ40 *Hs*Alg11	truncated human Alg11 (Δ1-40) cloned in MCS of pET28a-SIMPLEx; Kan^R	This study
pET28a-SIMPLEx-*Hs*Alg12	human Alg12 cloned in MCS of pET28a-SIMPLEx; Kan^R	This study
pET28a-SIMPLEx-*Hs*Alg13	human Alg13 cloned in MCS of pET28a-SIMPLEx; Kan^R	This study

pET28a-SIMPLEx-Δ24 *Hs*Alg14	truncated human Alg14 (Δ1-24) cloned in MCS of pET28a-SIMPLEx; Kan^R	This study
pET28a-SIMPLEx-*Hs*DPM1	human DPM1 cloned in MCS of pET28a-SIMPLEx; Kan^R	This study
pET28a-SIMPLEx-*Hs*PIGM	human PIGM cloned in MCS of pET28a-SIMPLEx; Kan^R	This study
pET28a-SIMPLEx-*Hs*PIGB	human PIGB cloned in MCS of pET28a-SIMPLEx; Kan^R	This study
pET28a-SIMPLEx-*Hs*PIGZ	human PIGZ cloned in MCS of pET28a-SIMPLEx; Kan^R	This study
pET28a-SIMPLEx-Δ28 *Hs*Alg5	truncated human Alg5 (Δ1-28) cloned in MCS of pET28a-SIMPLEx; Kan^R	This study
pET28a-SIMPLEx-*Hs*Alg6	human Alg6 cloned in MCS of pET28a-SIMPLEx; Kan^R	This study
pET28a-SIMPLEx-*Hs*Alg8	human Alg8 cloned in MCS of pET28a-SIMPLEx; Kan^R	This study
pET28a-SIMPLEx-*Hs*Alg10	human Alg10 cloned in MCS of pET28a-SIMPLEx; Kan^R	This study
pET28a-SIMPLEx-*Hs*UGCG	human UGCG cloned in MCS of pET28a-SIMPLEx; Kan^R	This study
pET28a-SIMPLEx-Δ27 *Hs*B3GLCT	truncated human B3GLCT (Δ1-27) cloned in MCS of pET28a-SIMPLEx; Kan^R	This study
pET28a-SIMPLEx-*Hs*GLYG	human Glycogenin cloned in MCS of pET28a-SIMPLEx; Kan^R	This study
pET28a-SIMPLEx-*Hs*POGLUT1	human POGLUT1 cloned in MCS of pET28a-SIMPLEx; Kan^R	This study
pET28a-SIMPLEx-Δ29*Hs*GnTI/MGAT1	truncated human GnTI (Δ1-29) cloned in MCS of pET28a-SIMPLEx; Kan^R	This study
pET28a-SIMPLEx-Δ29*Hs*GnTII/MGAT2	truncated human GnTII (Δ1-29) cloned in MCS of pET28a-SIMPLEx; Kan^R	This study
pET28a-SIMPLEx-Δ23*Hs*GnTIII/MGAT3	truncated human GnTIII (Δ1-23) cloned in MCS of pET28a-SIMPLEx; Kan^R	This study
pET28a-SIMPLEx-Δ27*Hs*GnTIV/MGAT4	truncated human GnTIV (Δ1-27) cloned in MCS of pET28a-SIMPLEx; Kan^R	This study
pET28a-SIMPLEx-Δ32*Hs*GCNT1	truncated human GCNT1 (Δ1-32) cloned in MCS of pET28a-SIMPLEx; Kan^R	This study
pET28a-SIMPLEx-Δ23*Hs*GCNT2	truncated human GCNT2 (Δ1-23) cloned in MCS of pET28a-SIMPLEx; Kan^R	This study
pET28a-SIMPLEx-Δ28*Hs*B3GNT2	truncated human B3GNT2 (Δ1-28) cloned in MCS of pET28a-SIMPLEx; Kan^R	This study
pET28a-SIMPLEx-Δ31*Hs*B3GNT6	truncated human B3GNT6 (Δ1-31) cloned in MCS of pET28a-SIMPLEx; Kan^R	This study
pET28a-SIMPLEx-*Hs*PIGA	human PIGA cloned in MCS of pET28a-SIMPLEx; Kan^R	This study
pET28a-SIMPLEx-Δ25*Hs*UGT1A1	truncated human UGT1A1 (Δ1-25) cloned in MCS of pET28a-SIMPLEx; Kan^R	This study
pET28a-SIMPLEx-Δ36*Hs*UGT1A3	truncated human UGT1A3 (Δ1-36) cloned in MCS of pET28a-SIMPLEx; Kan^R	This study
pET28a-SIMPLEx-Δ36*Hs*B4GAT1	truncated human B4GAT1 (Δ1-36) cloned in MCS of pET28a-SIMPLEx; Kan^R	This study
pET28a-SIMPLEx-Δ28*Hs*XXLT1	truncated human XXLT1 (Δ1-28) cloned in MCS of pET28a-SIMPLEx; Kan^R	This study
pET28a-SIMPLEx-*Cj*CstII	*C. jejuni* CstII cloned in MCS of pET28a-SIMPLEx; Kan^R	This study
pET28a-SIMPLEx-*Nm*Pst	*N. meningitidis* PolysiaT cloned in MCS of pET28a-SIMPLEx; Kan^R	This study
pET28a-SIMPLEx-*Cj*CgtB	*C. jejuni* CgtB cloned in MCS of pET28a-SIMPLEx; Kan^R	This study
pET28a-SIMPLEx-*Hp*LgtB	*H. pylori* GalT cloned in MCS of pET28a-SIMPLEx; Kan^R	This study
pET28a-SIMPLEx-*Nm*LgtB	*N. meningitidis* GalT cloned in MCS of pET28a-SIMPLEx; Kan^R	This study
pET28a-SIMPLEx-*Ng*LgtB	*N. gonorrhea* GalT cloned in MCS of pET28a-SIMPLEx; Kan^R	This study
pET28a-SIMPLEx-*Ec*WbgL	*E. coli* WbgL cloned in MCS of pET28a-SIMPLEx; Kan^R	This study
pET28a-SIMPLEx-*Ec*WecA	*E. coli* WecA cloned in MCS of pET28a-SIMPLEx; Kan^R	This study
pET28a-SIMPLEx-*Lp*SetA	*L. pneumophila* SetA cloned in MCS of pET28a-SIMPLEx; Kan^R	This study
pET28a-SIMPLEx-*Nm*SynE	*N. meningitidis* SynE cloned in MCS of pET28a-SIMPLEx; Kan^R	This study
pET28a-SIMPLEx-Δ34*Sc*Alg1	truncated *S. cerevisae* Alg1 (Δ1-34) cloned in MCS of pET28a-SIMPLEx; Kan^R	This study
pET28a-SIMPLEx-*Sc*Alg2	*S. cerevisae* Alg2 cloned in MCS of pET28a-SIMPLEx; Kan^R	This study
pET28a-SIMPLEx-Δ45*Sc*Alg11	truncated *S. cerevisae* Alg11 (Δ1-45) cloned in MCS of pET28a-SIMPLEx; Kan^R	This study
pET28a-SIMPLEx-Δ30*Nt*GnTI	truncated *N. tabacum* GnTI (Δ1-30) cloned in MCS of pET28a-SIMPLEx; Kan^R	This study
pET28a-SIMPLEx-Δ35*Nt*GnTII	truncated *N. tabacum* GnTII (Δ1-35) cloned in MCS of pET28a-SIMPLEx; Kan^R	This study
pET28a-SIMPLEx-Δ22*Bt*GGTA1	truncated *B. taurus* GGTA1 (Δ1-35) cloned in MCS of pET28a-SIMPLEx; Kan^R	This study
pET28a-SIMPLEx-Δ60*Mm*GGTA1	truncated *M. musculus* GGTA1 (Δ1-60) cloned in MCS of pET28a-SIMPLEx; Kan^R	This study

pET28a-SIMPLEx-Δ58*Rn*GGTA1	truncated *R. norvergicus* GGTA1 (Δ1-58) cloned in MCS of pET28a-SIMPLEx; Kan^R	This study
pET28a-SIMPLEx-Δ44*Bt*B4GalT1	truncated *B. taurus* B4GalT1 (Δ1-35) cloned in MCS of pET28a-SIMPLEx; Kan^R	This study
pET28a-SIMPLEx-*Hs*CDK4	human cyclin-dependent kinase 4 (CDK4) cloned in MCS of pET28a-SIMPLEx; Kan^R	This study
pET28a-SIMPLEx-*Hs*CDKN2A	human cyclin-dependent kinase inhibitor 2A (CDKN2A) cloned in MCS of pET28a-SIMPLEx; Kan^R	This study
pET28a-SIMPLEx-*Hs*EGFR_TK	truncated human EGFR (P694-G1022) cloned in MCS of pET28a-SIMPLEx; Kan^R	This study
pET28a-SIMPLEx-*Hs*FOS	human proto-oncogene c-Fos cloned in MCS of pET28a-SIMPLEx; Kan^R	This study
pET28a-SIMPLEx-*Hs*GATA2	human endothelial transcription factor GATA cloned in MCS of pET28a-SIMPLEx; Kan^R	This study
pET28a-SIMPLEx-*Hs*JUN	human transcription factor JUN cloned in MCS of pET28a-SIMPLEx; Kan^R	This study
pET28a-SIMPLEx-*Hs*MMP1	human interstitial collagenase cloned in MCS of pET28a-SIMPLEx; Kan^R	This study
pET28a-SIMPLEx-*Hs*Proinsulin	human proinsulin cloned in MCS of pET28a-SIMPLEx; Kan^R	This study
pcDNA3-SIMPLEx-Δ26*Hs*ST6Gal1	truncated human ST6Gal1 (Δ1-26) as SIMPLEx chimera cloned in pcDNA3; Amp^R	This study
pYS338-SIMPLEx-Δ26*Hs*ST6Gal1	truncated human ST6Gal1 (Δ1-26) as SIMPLEx chimera cloned in pYS338; Amp^R	This study
pJL1-SIMPLEx-Δ26*Hs*ST6Gal1	truncated human ST6Gal1 (Δ1-26) as SIMPLEx chimera cloned in pJL1; Kan^R	This study
pET28a-Δ25*Hs*FUT1	truncated human FUT1 (Δ1-25) with C-terminal 6xHis tag in pET28a(+); Kan^R	This study
pET28a-Δ28*Hs*FUT2	truncated human FUT2 (Δ1-28) with C-terminal 6xHis tag in pET28a(+); Kan^R	This study
pET28a-Δ34*Hs*FUT3	truncated human FUT3 (Δ1-34) with C-terminal 6xHis tag in pET28a(+); Kan^R	This study
pET28a-Δ172*Hs*FUT4	truncated human FUT4 (Δ1-172) with C-terminal 6xHis tag in pET28a(+); Kan^R	This study
pET28a-Δ34*Hs*FUT5	truncated human FUT5 (Δ1-34) with C-terminal 6xHis tag in pET28a(+); Kan^R	This study
pET28a-Δ34*Hs*FUT6	truncated human FUT6 (Δ1-34) with C-terminal 6xHis tag in pET28a(+); Kan^R	This study
pET28a-Δ36*Hs*FUT7	truncated human FUT7 (Δ1-36) with C-terminal 6xHis tag in pET28a(+); Kan^R	This study
pET28a-Δ30*Hs*FUT8	truncated human FUT8 (Δ1-30) with C-terminal 6xHis tag in pET28a(+); Kan^R	This study
pET28a-Δ32*Hs*FUT9	truncated human FUT9 (Δ1-32) with C-terminal 6xHis tag in pET28a(+); Kan^R	This study
pET28a-Δ31*Hs*FUT10	truncated human FUT10 (Δ1-31) with C-terminal 6xHis tag in pET28a(+); Kan^R	This study
pET28a-Δ24*Hs*FUT11	truncated human FUT11 (Δ1-24) with C-terminal 6xHis tag in pET28a(+); Kan^R	This study
pET28a-Δ26*Hs*POFUT1	truncated human POFUT1 (Δ1-26) with C-terminal 6xHis tag in pET28a(+); Kan^R	This study
pET28a-Δ34*Hs*ST3Gal1	truncated human ST3Gal1 (Δ1-34) with C-terminal 6xHis tag in pET28a(+); Kan^R	This study
pET28a-Δ28*Hs*ST3Gal3	truncated human ST3Gal3 (Δ1-28) with C-terminal 6xHis tag in pET28a(+); Kan^R	This study
pET28a-Δ26*Hs*ST3Gal4	truncated human ST3Gal4 (Δ1-26) with C-terminal 6xHis tag in pET28a(+); Kan^R	This study
pET28a-Δ25*Hs*ST3Gal6	truncated human ST3Gal6 (Δ1-25) with C-terminal 6xHis tag in pET28a(+); Kan^R	This study
pET28a-Δ26*Hs*ST6Gal1	truncated human ST6Gal1 (Δ1-26) with C-terminal 6xHis tag in pET28a(+); Kan^R	This study
pET28a-*Hs*ST6Gal1	Human ST6Gal1 with C-terminal 6xHis tag in pET28a(+); Kan^R	This study
pET28a-MBP-Δ26*Hs*ST6Gal1	truncated human ST6Gal1 (Δ1-26) with C-terminal 6xHis tag in pET28a(+); Kan^R	This study
pET28a-Δ26*Hs*ST6Gal1-ApoAI	truncated human ST6Gal1 (Δ1-26) with ApoAI*-6xHis in pET28a(+); Kan^R	This study
pET28a-Δ35*Hs*ST6GalNAc1	truncated human ST6GalNAc1 (Δ1-35) with C-terminal 6xHis tag in pET28a(+); Kan^R	This study
pET28a-Δ28*Hs*ST6GalNAc2	truncated human ST6GalNAc2 (Δ1-28) with C-terminal 6xHis tag in pET28a(+); Kan^R	This study
pET28a-Δ27*Hs*ST6GalNAc4	truncated human ST6GalNAc4 (Δ1-27) with C-terminal 6xHis tag in pET28a(+); Kan^R	This study
pET28a-Δ48*Hs*ST8Sia1	truncated human ST8Sia1 (Δ1-48) with C-terminal 6xHis tag in pET28a(+); Kan^R	This study
pET28a-Δ23*Hs*ST8Sia2	truncated human ST8Sia2 (Δ1-23) with C-terminal 6xHis tag in pET28a(+); Kan^R	This study

pET28a-Δ33*Hs*ST8Sia3	truncated human ST8Sia3 (Δ1-33) with C-terminal 6xHis tag in pET28a(+); Kan^R	This study
pET28a-Δ20*Hs*ST8Sia4	truncated human ST8Sia4 (Δ1-20) with C-terminal 6xHis tag in pET28a(+); Kan^R	This study
pET28a-Δ28*Hs*ppGalNAcT1	truncated human ppGalNAcT1 (Δ1-28) with C-terminal 6xHis tag in pET28a(+); Kan^R	This study
pET28a-Δ24*Hs*ppGalNAcT2	truncated human ppGalNAcT2 (Δ1-24) with C-terminal 6xHis tag in pET28a(+); Kan^R	This study
pET28a-Δ37*Hs*ppGalNAcT3	truncated human ppGalNAcT3 (Δ1-37) with C-terminal 6xHis tag in pET28a(+); Kan^R	This study
pET28a-Δ35*Hs*ppGalNAcT4	truncated human ppGalNAcT4 (Δ1-35) with C-terminal 6xHis tag in pET28a(+); Kan^R	This study
pET28a-Δ35*Hs*ppGalNAcT5	truncated human ppGalNAcT5 (Δ1-35) with C-terminal 6xHis tag in pET28a(+); Kan^R	This study
pET28a-Δ28*Hs*ppGalNAcT6	truncated human ppGalNAcT6 (Δ1-28) with C-terminal 6xHis tag in pET28a(+); Kan^R	This study
pET28a-Δ29*Hs*ppGalNAcT7	truncated human ppGalNAcT7 (Δ1-29) with C-terminal 6xHis tag in pET28a(+); Kan^R	This study
pET28a-Δ29*Hs*ppGalNAcT8	truncated human ppGalNAcT8 (Δ1-29) with C-terminal 6xHis tag in pET28a(+); Kan^R	This study
pET28a-Δ28*Hs*ppGalNAcT9	truncated human ppGalNAcT9 (Δ1-28) with C-terminal 6xHis tag in pET28a(+); Kan^R	This study
pET28a-Δ31*Hs*ppGalNAcT10	truncated human ppGalNAcT10 (Δ1-31) with C-terminal 6xHis tag in pET28a(+); Kan^R	This study
pET28a-Δ43*Hs*B3GALNT1	truncated human B3GALNT1 (Δ1-43) with C-terminal 6xHis tag in pET28a(+); Kan^R	This study
pET28a-Δ25*Hs*B4GALNT1	truncated human B4GALNT1 (Δ1-25) with C-terminal 6xHis tag in pET28a(+); Kan^R	This study
pET28a-*Hs*-A-group	human A-ABO with C-terminal 6xHis tag in pET28a(+); Kan^R	This study
pET28a-Δ43*Hs*A4GALT	truncated human A4GALT (Δ1-43) with C-terminal 6xHis tag in pET28a(+); Kan^R	This study
pET28a-Δ26*Hs*B3GalT1	truncated human B3GalT1 (Δ1-26) with C-terminal 6xHis tag in pET28a(+); Kan^R	This study
pET28a-Δ45*Hs*B3GalT2	truncated human B3GalT2 (Δ1-45) with C-terminal 6xHis tag in pET28a(+); Kan^R	This study
pET28a-Δ44*Hs*B4GalT1	truncated human B4GalT1 (Δ1-44) with C-terminal 6xHis tag in pET28a(+); Kan^R	This study
pET28a-Δ36*Hs*B4GalT2	truncated human B4GalT2 (Δ1-36) with C-terminal 6xHis tag in pET28a(+); Kan^R	This study
pET28a-Δ31*Hs*B4GalT3	truncated human B4GalT3 (Δ1-31) with C-terminal 6xHis tag in pET28a(+); Kan^R	This study
pET28a-Δ38*Hs*B4GalT4	truncated human B4GalT4 (Δ1-38) with C-terminal 6xHis tag in pET28a(+); Kan^R	This study
pET28a-Δ35*Hs*B4GalT5	truncated human B4GalT5 (Δ1-35) with C-terminal 6xHis tag in pET28a(+); Kan^R	This study
pET28a-Δ35*Hs*B4GalT6	truncated human B4GalT6 (Δ1-35) with C-terminal 6xHis tag in pET28a(+); Kan^R	This study
pET28a-*Hs*-B-group	human B-ABO with C-terminal 6xHis tag in pET28a(+); Kan^R	This study
pET28a-Δ20*Hs*UGT8	truncated human UGT8 (Δ1-20) with C-terminal 6xHis tag in pET28a(+); Kan^R	This study
pET28a-Δ29*Hs*C1GLT	truncated human C1GLT (Δ1-29) with C-terminal 6xHis tag in pET28a(+); Kan^R	This study
pET28a-*Hs*COSMC	human COSMC with C-terminal 6xHis tag in pET28a(+); Kan^R	This study
pET28a-Δ23*Hs*Alg1	truncated human Alg1 (Δ1-23) with C-terminal 6xHis tag in pET28a(+); Kan^R	This study
pET28a-*Hs*Alg2	human Alg2 with C-terminal 6xHis tag in pET28a(+); Kan^R	This study
pET28a-*Hs*Alg3	human Alg3 with C-terminal 6xHis tag in pET28a(+); Kan^R	This study
pET28a-Δ40*Hs*Alg11	truncated human Alg11 (Δ1-40) with C-terminal 6xHis tag in pET28a(+); Kan^R	This study
pET28a-*Hs*Alg12	human Alg12 with C-terminal 6xHis tag in pET28a(+); Kan^R	This study
pET28a-*Hs*Alg13	human Alg13 with C-terminal 6xHis tag in pET28a(+); Kan^R	This study
pET28a-Δ24*Hs*Alg14	truncated human Alg14 (Δ1-24) with C-terminal 6xHis tag in pET28a(+); Kan^R	This study
pET28a-*Hs*DPM1	human DPM1 with C-terminal 6xHis tag in pET28a(+); Kan^R	This study
pET28a-*Hs*PIGM	human PIGM with C-terminal 6xHis tag in pET28a(+); Kan^R	This study

pET28a-*Hs*PIGB	human PIGB with C-terminal 6xHis tag in pET28a(+); Kan^R	This study
pET28a-*Hs*PIGZ	human PIGZ with C-terminal 6xHis tag in pET28a(+); Kan^R	This study
pET28a-Δ28*Hs*Alg5	truncated human Alg5 (Δ1-28) with C-terminal 6xHis tag in pET28a(+); Kan^R	This study
pET28a-*Hs*Alg6	human Alg6 with C-terminal 6xHis tag in pET28a(+); Kan^R	This study
pET28a-*Hs*Alg8	human Alg8 with C-terminal 6xHis tag in pET28a(+); Kan^R	This study
pET28a-*Hs*Alg10	human Alg10 with C-terminal 6xHis tag in pET28a(+); Kan^R	This study
pET28a-*Hs*UGCG	human UGCG with C-terminal 6xHis tag in pET28a(+); Kan^R	This study
pET28a-Δ27*Hs*B3GLCT	truncated human B3GLCT (Δ1-27) with C-terminal 6xHis tag in pET28a(+); Kan^R	This study
pET28a-*Hs*GLYG	human glycogenin with C-terminal 6xHis tag in pET28a(+); Kan^R	This study
pET28a-*Hs*POGLUT1	human POGLUT1 with C-terminal 6xHis tag in pET28a(+); Kan^R	This study
pET28a-Δ29*Hs*GnTI/MGAT1	truncated human GnTI (Δ1-29) with C-terminal 6xHis tag in pET28a(+); Kan^R	This study
pET28a-Δ29*Hs*GnTII/MGAT2	truncated human GnTII (Δ1-29) with C-terminal 6xHis tag in pET28a(+); Kan^R	This study
pET28a-Δ23*Hs*GnTIII/MGAT3	truncated human GnTIII (Δ1-23) with C-terminal 6xHis tag in pET28a(+); Kan^R	This study
pET28a-Δ27*Hs*GnTIV/MGAT4	truncated human GnTIV (Δ1-27) with C-terminal 6xHis tag in pET28a(+); Kan^R	This study
pET28a-Δ32*Hs*GCNT1	truncated human GCNT1 (Δ1-32) with C-terminal 6xHis tag in pET28a(+); Kan^R	This study
pET28a-Δ23*Hs*GCNT2	truncated human GCNT2 (Δ1-23) with C-terminal 6xHis tag in pET28a(+); Kan^R	This study
pET28a-Δ28*Hs*B3GNT2	truncated human B3GnT2 (Δ1-28) with C-terminal 6xHis tag in pET28a(+); Kan^R	This study
pET28a-Δ31*Hs*B3GNT6	truncated human B3GnT6 (Δ1-31) with C-terminal 6xHis tag in pET28a(+); Kan^R	This study
pET28a-*Hs*PIGA	human PIGA with C-terminal 6xHis tag in pET28a(+); Kan^R	This study
pET28a-Δ25*Hs*UGT1A1	truncated human UGT1A1 (Δ1-25) with C-terminal 6xHis tag in pET28a(+); Kan^R	This study
pET28a-Δ36*Hs*UGT1A3	truncated human UGT1A3 (Δ1-36) with C-terminal 6xHis tag in pET28a(+); Kan^R	This study
pET28a-Δ36*Hs*B4GAT1	truncated human B4GAT1 (Δ1-36) with C-terminal 6xHis tag in pET28a(+); Kan^R	This study
pET28a-Δ28*Hs*XXLT1	truncated human XXLT1 (Δ1-28) with C-terminal 6xHis tag in pET28a(+); Kan^R	This study
pET28a-*Cj*CstII	*C. jejuni* CstII with C-terminal 6xHis tag in pET28a(+); Kan^R	This study
pET28a-*Nm*Pst	*N. meningitidis* PolysiaT with C-terminal 6xHis tag in pET28a(+); Kan^R	This study
pET28a-*Cj*CgtB	*C. jejuni* CgtB with C-terminal 6xHis tag in pET28a(+); Kan^R	This study
pET28a-*Hp*LgtB	*H. pylori* GalT with C-terminal 6xHis tag in pET28a(+); Kan^R	This study
pET28a-*Nm*LgtB	*N. meningitidis* GalT with C-terminal 6xHis tag in pET28a(+); Kan^R	This study
pET28a-*Ng*LgtB	*N. gonorrhea* GalT with C-terminal 6xHis tag in pET28a(+); Kan^R	This study
pET28a-*Ec*WbgL	*E. coli* WbgL with C-terminal 6xHis tag in pET28a(+); Kan^R	This study
pET28a-*Ec*WecA	*E. coli* WecA with C-terminal 6xHis tag in pET28a(+); Kan^R	This study
pET28a-*Lp*SetA	*L. pneumophila* SetA with C-terminal 6xHis tag in pET28a(+); Kan^R	This study
pET28a-*Nm*SynE	*N. meningitidis* SynE with C-terminal 6xHis tag in pET28a(+); Kan^R	This study
pET28a-Δ34*Sc*Alg1	truncated *S. cerevisae* Alg1 (Δ1-34) with C-terminal 6xHis tag in pET28a(+); Kan^R	This study
pET28a-*Sc*Alg2	*S. cerevisae* Alg2 with C-terminal 6xHis tag in pET28a(+); Kan^R	This study
pET28a-Δ45*Sc*Alg11	truncated *S. cerevisae* Alg11 (Δ1-45) with C-terminal 6xHis tag in pET28a(+); Kan^R	This study
pET28a-Δ30*Nt*GnTI	truncated *N. tabacum* GnTI (Δ1-30) with C-terminal 6xHis tag in pET28a(+); Kan^R	This study
pET28a-Δ35*Nt*GnTII	truncated *N. tabacum* GnTII (Δ1-35) with C-terminal 6xHis tag in pET28a(+); Kan^R	This study
pET28a-Δ22*Bt*GGTA1	truncated *B. taurus* GGTA1 (Δ1-35) with C-terminal 6xHis tag in pET28a(+); Kan^R	This study
pET28a-Δ60*Mm*GGTA1	truncated *M. musculus* GGTA1 (Δ1-60) with C-terminal 6xHis tag in pET28a(+); Kan^R	This study

pET28a-Δ58*Rn*GGTA1	truncated *R. norvergicus* GGTA1 (Δ1-58) with C-terminal 6xHis tag in pET28a(+); Kan^R	This study
pET28a-Δ44*Bt*B4GalT1	truncated *B. taurus* B4GalT1 (Δ1-35) with C-terminal 6xHis tag in pET28a(+); Kan^R	This study
pET28a-*Hs*CDK4	human CDK4 with C-terminal 6xHis tag in pET28a(+); Kan^R	This study
pET28a-*Hs*CDKN2A	human CDKN2A with C-terminal 6xHis tag in pET28a(+); Kan^R	This study
pET28a-*Hs*EGFR_TK	truncated human EGFR (P694-G1022) with C-terminal 6xHis tag in pET28a(+); Kan^R	This study
pET28a-*Hs*FOS	human proto-oncogene c-Fos with C-terminal 6xHis tag in pET28a(+); Kan^R	This study
pET28a-*Hs*GATA2	human endothelial transcription factor GATA with C-terminal 6xHis tag in pET28a(+); Kan^R	This study
pET28a-*Hs*JUN	human transcription factor JUN with C-terminal 6xHis tag in pET28a(+); Kan^R	This study
pET28a-*Hs*MMP1	human Interstitial collagenase with C-terminal 6xHis tag in pET28a(+); Kan^R	This study
pET28a-*Hs*Pronsulin	human proinsulin with C-terminal 6xHis tag in pET28a(+); Kan^R	This study
pcDNA3-Δ26*Hs*ST6Gal1	truncated *Hs*ST6Gal1 with C-terminal 6xHis tag in mammalian expression vector pcDNA3; Amp^R	This study
pYS338-Δ26*Hs*ST6Gal1	truncated *Hs*ST6Gal1 with C-terminal 6xHis tag in yeast expression vector pYS338; Amp^R	This study
pJL1-Δ26*Hs*ST6Gal1	truncated *Hs*ST6Gal1 with C-terminal 6xHis tag in cell-free expression vector pJL1; Kan^R	This study
pConYCGmCB	Plasmid pMW07 encoding genes for $Man_3GlcNAc_2$ biosynthesis; Cm^R	Reference [2]
pTrc-spDsbA-MBP-GCG^{DQNAT}	Plasmid pTrc99Y encoding spDsbA-MBP-GCG^{DQNAT}; Amp^R	Reference [2]
pMAF10	Plasmid pMLBAD encoding HA-tagged PglB from *Campylobacter jejuni*, Tmp^R	Reference [7]

Supplementary Table 2. *N*-glycan structures produced in this study.

Glycan Number	Symbolic Representation	Oxford Notation	Structure
**1**	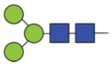	M3	Manα1-6(Manα1-3)Manβ1-4GlcNAcβ1-4GlcNAc
**2**	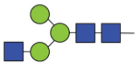	A1	Manα1-6(GlcNAcβ1-2Manα1-3)Manβ1-4GlcNAc β1-4GlcNAc
**3**	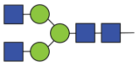	A2	GlcNAcβ1-2Manα1-6(GlcNAcβ1-2Manα1-3)Man β1-4GlcNAcβ1-4GlcNAc
**4**	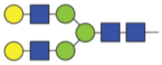	A2G2	Galβ1-4GlcNAcβ1-2Manα1-6(Galβ1-4GlcNAcβ1-2 Manα1-3)Manβ1-4GlcNAcβ1-4GlcNAc
**5**	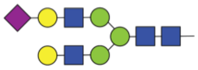	A2G2S1	Galβ1-4GlcNAcβ1-2Manα1-6(Neu5Acα2-6Galβ1-4GlcNAcβ1-2Manα1-3)Manβ1-4GlcNAcβ1-4 GlcNAc
**6**	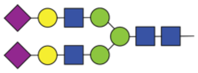	A2G2S2	Neu5Acα2-6Galβ1-4GlcNAcβ1-2Manα1-6 (Neu5Acα2-6Galβ1-4GlcNAcβ1-2Manα1-3) Manβ1-4GlcNAcβ1-4GlcNAc
**7**	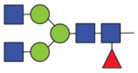	FA2	GlcNAcβ1-2Manα1-6(GlcNAcβ1-2Manα1-3) Manβ1-4GlcNAcβ1-4(Fucα1-6)GlcNAc
**8**	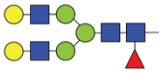	FA2G2	Galβ1-4GlcNAcβ1-2Manα1-6(Galβ1-4GlcNAcβ1-2 Manα1-3)Manβ1-4GlcNAcβ1-4(Fucα1-6)GlcNAc
**9**	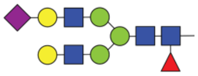	FA2G2S1	Galβ1-4GlcNAcβ1-2Manα1-6(Neu5Acα2-6Galβ1-4GlcNAcβ1-2Manα1-3)Manβ1-4GlcNAcβ1-4 (Fucα1-6)GlcNAc
**10**	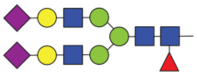	FA2G2S2	Neu5Acα2-6Galβ1-4GlcNAcβ1-2Manα1-6 (Neu5Acα2-6Galβ1-4GlcNAcβ1-2Manα1-3) Manβ1-4(Fucα1-6)GlcNAcβ1-4GlcNAc
**11**	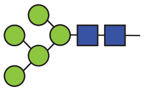	M5	Manα1-6(Manα1-3)Manα1-6(Manα1-3)Manβ1-4GlcNAcβ1-4GlcNAc

**12**	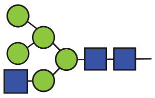	M5A1	Manα1-6(Manα1-3)Manα1-6(GlcNAcβ1-2Manα1-3)Manβ1-4GlcNAcβ1-4GlcNAc
**13**	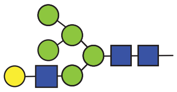	M5A1G1	Manα1-6(Manα1-3)Manα1-6(Galβ1-4GlcNAcβ1-2Manα1-3)Manβ1-4GlcNAcβ1-4GlcNAc
**14**	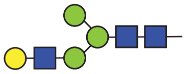	A1G1	Manα1-6(Galβ1-4GlcNAcβ1-2Manα1-3)Manβ1-4GlcNAcβ1-4GlcNAc
**15**	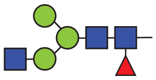	FA1	Manα1-6(GlcNAcβ1-2Manα1-3)Manβ1-4 GlcNAc β1-4(Fucα1-6)GlcNAc
**16**	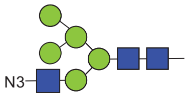	N/A	Manα1-6(Manα1-3)Manα1-6(GlcNAzβ1-2Manα1-3)Manβ1-4GlcNAcβ1-4GlcNAc
**17**	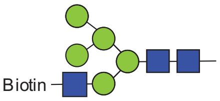	N/A	Manα1-6(Manα1-3)Manα1-6(Biotin-PEG_4-DBCO-GlcNAzβ1-2Manα1-3)Manβ1-4GlcNAcβ1-4GlcNAc
**18**	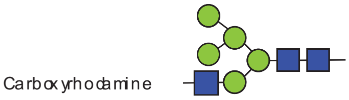	N/A	Manα1-6(Manα1-3)Manα1-6(CR110-DBCO-GlcNAzβ1-2Manα1-3)Manβ1-4GlcNAcβ1-4GlcNAc

Supplementary References

1. Dyson, M.R. et al. Production of soluble mammalian proteins in *Escherichia coli*: identification of protein features that correlate with successful expression. *BMC Biotechnol* 4, 32 (2004).
2. Glasscock, C.J. *et al*. A flow cytometric approach to engineering *Escherichia coli* for improved eukaryotic protein glycosylation. *Metab Eng* 47, 488-495 (2018).
3. Hamilton, B.S. *et al*. A library of chemically defined human *N*-glycans synthesized from microbial oligosaccharide precursors. *Sci Rep* 7, 15907 (2017).
4. Mizrachi, D. *et al*. Making water-soluble integral membrane proteins in vivo using an amphipathic protein fusion strategy. *Nat Commun* 6, 6826 (2015).
5. Stark, J.C. *et al*. BioBits Bright: A fluorescent synthetic biology education kit. *Sci Adv* 4, eaat5107 (2018).
6. Dodev, T.S. *et al.* A tool kit for rapid cloning and expression of recombinant antibodies. *Sci Rep* 4, 5885 (2014).
7. Feldman, M.F. *et al*. Engineering *N*-linked protein glycosylation with diverse O antigen lipopolysaccharide structures in *Escherichia coli*. *Proc Natl Acad Sci U S A* 102, 3016-21 (2005).
