# Supplementary material for: A universal glycoenzyme biosynthesis pipeline that enables efficient cell-free remodeling of glycans
Source: Nat Commun. 2022 Oct 24;13:6325. doi: 10.1038/s41467-022-34029-7 (PMC9592599; doi:10.1038/s41467-022-34029-7)
Supplement: Supplementary file 3 — Description of Supplementary Information [file 41467_2022_34029_MOESM3_ESM.pdf]

Description of Supporting Information

- **Supplementary Dataset 1** – an Excel spreadsheet containing detailed information about each of the GTs that were evaluated in the main manuscript.
