# Supplementary material for: A universal glycoenzyme biosynthesis pipeline that enables efficient cell-free remodeling of glycans
Source: Nat Commun. 2022 Oct 24;13:6325. doi: 10.1038/s41467-022-34029-7 (PMC9592599; doi:10.1038/s41467-022-34029-7)
Supplement: Supplementary file 5 — Reporting Summary [file 41467_2022_34029_MOESM5_ESM.pdf]

nature portfolio

Corresponding author(s): Matthew DeLisa

Last updated by author(s): Sep 15, 2022

Reporting Summary

Nature Portfolio wishes to improve the reproducibility of the work that we publish. This form provides structure for consistency and transparency in reporting. For further information on Nature Portfolio policies, see our Editorial Policies and the Editorial Policy Checklist.

Statistics

For all statistical analyses, confirm that the following items are present in the figure legend, table legend, main text, or Methods section.

n/a	Confirmed	
☐	☒	The exact sample size (n) for each experimental group/condition, given as a discrete number and unit of measurement
☐	☒	A statement on whether measurements were taken from distinct samples or whether the same sample was measured repeatedly
☐	☒	The statistical test(s) used AND whether they are one- or two-sided<br>*Only common tests should be described solely by name; describe more complex techniques in the Methods section.*
☒	☐	A description of all covariates tested
☒	☐	A description of any assumptions or corrections, such as tests of normality and adjustment for multiple comparisons
☐	☒	A full description of the statistical parameters including central tendency (e.g. means) or other basic estimates (e.g. regression coefficient) AND variation (e.g. standard deviation) or associated estimates of uncertainty (e.g. confidence intervals)
☐	☒	For null hypothesis testing, the test statistic (e.g. F, t, r) with confidence intervals, effect sizes, degrees of freedom and P value noted<br>*Give P values as exact values whenever suitable.*
☒	☐	For Bayesian analysis, information on the choice of priors and Markov chain Monte Carlo settings
☒	☐	For hierarchical and complex designs, identification of the appropriate level for tests and full reporting of outcomes
☒	☐	Estimates of effect sizes (e.g. Cohen's d, Pearson's r), indicating how they were calculated

Our web collection on statistics for biologists contains articles on many of the points above.

Software and code

Policy information about availability of computer code

Data collection	Image Lab 6.1 software (Bio-Rad) was used for collecting Western blot images.
Data analysis	Image Lab 6.1 software (Bio-Rad) was used for visualizing/analyzing Western blots. All MS and MS/MS raw spectra from each sample obtained by MRM-HR scan were analyzed by SCIEX OS 1.4 data analysis system. Microsoft Excel, Prism 9 for MacOS version 9.2.0, or R version 3.4.2 software was used for generating graphs and analyzing data collected in all experiments.

For manuscripts utilizing custom algorithms or software that are central to the research but not yet described in published literature, software must be made available to editors and reviewers. We strongly encourage code deposition in a community repository (e.g. GitHub). See the Nature Portfolio guidelines for submitting code & software for further information.

Data

Policy information about availability of data

All manuscripts must include a data availability statement. This statement should provide the following information, where applicable:

- Accession codes, unique identifiers, or web links for publicly available datasets
- A description of any restrictions on data availability
- For clinical datasets or third party data, please ensure that the statement adheres to our policy

All data generated or analyzed during this study are included in this article and its Supplementary Information/Source Data file that are provided with this paper.

Human research participants

Policy information about studies involving human research participants and Sex and Gender in Research.

Reporting on sex and gender	N/A
Population characteristics	N/A
Recruitment	N/A
Ethics oversight	N/A

Note that full information on the approval of the study protocol must also be provided in the manuscript.

Field-specific reporting

Please select the one below that is the best fit for your research. If you are not sure, read the appropriate sections before making your selection.

[x] Life sciences [ ] Behavioural & social sciences [ ] Ecological, evolutionary & environmental sciences

For a reference copy of the document with all sections, see nature.com/documents/nr-reporting-summary-flat.pdf

Life sciences study design

All studies must disclose on these points even when the disclosure is negative.

Sample size	Sample sizes were not predetermined based on statistical methods, but were chosen according to the standards of the field (at least three independent biological replicates for each condition), which gave sufficient statistics for the effect sizes of interest.
Data exclusions	No data were excluded from the analyses.
Replication	To ensure robust reproducibility of all results presented in the paper, we performed three biological replicates of each. In every experiment presented, the results were found to be reproducible.
Randomization	The experiments were not randomized. All samples were analyzed equally with no sub-sampling and thus there was no requirement for randomization.
Blinding	Investigators were not blinded. Blinding during collection was not needed because conditions were well controlled. Blinding during analysis was not feasible as the differences between samples under different conditions were visually apparent in the collected data. Blinding is also not necessary because the results are quantitative and did not require subjective judgment or interpretation. Blinding is not typically used in the field.

Reporting for specific materials, systems and methods

We require information from authors about some types of materials, experimental systems and methods used in many studies. Here, indicate whether each material, system or method listed is relevant to your study. If you are not sure if a list item applies to your research, read the appropriate section before selecting a response.

Materials & experimental systems

n/a	Involved in the study	
[ ]	[x]	Antibodies
[ ]	[x]	Eukaryotic cell lines
[x]	[ ]	Palaeontology and archaeology
[x]	[ ]	Animals and other organisms
[x]	[ ]	Clinical data
[x]	[ ]	Dual use research of concern

Methods

n/a	Involved in the study	
[x]	[ ]	ChIP-seq
[x]	[ ]	Flow cytometry
[x]	[ ]	MRI-based neuroimaging

Antibodies

Antibodies used	1. rabbit polyclonal antibody to 6xHis epitope tag (Thermo Fisher Scientific # PA1-983B; 1:5,000 dilution)<br>2. mouse anti-GAPDH clone 6C5 (Calbiochem # CB1001; 1:10,000 dilution)<br>3. rabbit polyclonal anti-GroEL (Sigma # G6532; 1:20,000 dilution)

	4. rabbit anti-alpha tubulin clone EPR13799 (Abcam # ab184970; 1:10,000 dilution)<br>5. goat anti-rabbit IgG H&L (HRP) (Abcam # ab6721; 1:5,000 dilution)<br>6. rabbit anti-mouse IgG H&L (HRP) (Abcam # ab6728; 1:5,000 dilution)<br>7. ExtrAvidin®–Peroxidase (Sigma # E2886; 1:4,000 dilution)
Validation	All antibodies used in this work were comprehensively validated for quality and performance (specificity, sensitivity, cross-reactivity) as discussed on the vendor websites . Detailed protocols for usage of all of these antibodies can be found at the following websites:<br>1. https://www.thermofisher.com/antibody/product/6x-His-Tag-Antibody-Polyclonal/PA1-983B<br>2. https://www.sigmaaldrich.com/US/en/product/mm/cb1001<br>3. https://www.sigmaaldrich.com/US/en/product/sigma/g6532<br>4. https://www.abcam.com/alpha-tubulin-antibody-epr13799-ab184970.html<br>5. https://www.abcam.com/goat-rabbit-igg-hl-hrp-ab6721.html<br>6. https://www.abcam.com/rabbit-mouse-igg-hl-hrp-ab6728.html<br>7. https://www.sigmaaldrich.com/US/en/product/sigma/e2886

Eukaryotic cell lines

Policy information about cell lines and Sex and Gender in Research

Cell line source(s)	1. HEK293T (ATCC; CRL-3216)<br>2. FreeStyleTM 293-F cells (HEK293F; Thermo Fisher Scientific; catalog # R79007)<br>3. Expi293F cells (HEK293F GnTI- Thermo Fisher Scientific; catalog # A39240)
Authentication	Techniques/procedures for authenticating each cell line used in this study included morphology analysis, PCR assays with species-specific primers, and STR profiling, the latter of which was performed using ATCC's human cell STR profiling service.<br><br>Vendor's authentication details can be found at the following websites:<br>1. https://www.atcc.org/products/crl-3216<br>2. https://www.thermofisher.com/order/catalog/product/R79007<br>3. https://www.thermofisher.com/order/catalog/product/A39240
Mycoplasma contamination	All cell lines used in this study were confirmed to be negative for mycoplasma contamination.
Commonly misidentified lines<br>(See ICLAC register)	No commonly misidentified cell lines were used in the study.
